# Acetylation of the Mitochondrial Chaperone GRP75 Governs ER‐Mitochondrial Calcium Homeostasis and Hepatocyte Insulin Resistance

**DOI:** 10.1002/advs.202508991

**Published:** 2025-09-26

**Authors:** Danni Wang, Jiaqi Zhang, Xinyu Yang, Qiqi Zhang, Xiuya Hu, Xin Lu, Hanni Li, Xue Bai, Kai Zhang, Michael N. Sack, Yongsheng Chang, Yingmei Wang, Lingdi Wang, Lu Zhu

**Affiliations:** ^1^ Department of Pharmacology State Key Laboratory of Experimental Hematology Tianjin Key Laboratory of Inflammatory Biology The province and ministry co‐sponsored collaborative innovation center for medical epigenetics NHC Key Laboratory of Hormones and Development Chu Hsien‐I Memorial Hospital, and Tianjin Institute of Endocrinology School of Basic Medical Sciences Tianjin Medical University Tianjin 300070 China; ^2^ Department of Physiology and Pathophysiology Tianjin Key Laboratory of Cell Homeostasis and Major Diseases School of Basic Medical Sciences Tianjin Medical University Tianjin 300070 China; ^3^ Department of Biochemistry and Molecular Biology School of Basic Medical Sciences Tianjin Medical University Tianjin 300070 China; ^4^ Laboratory of Mitochondrial Biology and Metabolism NHLBI National Institutes of Health Bethesda MD 20892 USA; ^5^ Department of Gynecology and Obstetrics Tianjin Key Laboratory of Female Reproductive Health and Eugenics Tianjin Medical University General Hospital Tianjin 300052 China

**Keywords:** acetylation, insulin resistance, liver, metabolism, mitochondria

## Abstract

Overnutrition exacerbates insulin resistance (IR) and is linked to excessive mitochondrial protein acetylation. However, the molecular mechanism by which mitochondrial protein acetylation influences hepatic IR remains incompletely elucidated. To investigate this biology, GCN5L1 liver knockout mice (LKO), which exhibit blunted mitochondrial protein acetylation are utilized. Interestingly, the hepatocytes of LKO mice exhibit impaired insulin signaling and exaggerated endoplasmic reticulum (ER) stress. To explore putative mechanisms, protein‐interaction and acetyl‐proteome analyses are conducted following hepatic induction of GCN5L1. The mitochondrial chaperone GRP75 interacts with GCN5L1 and is acetylated on lysine residues K567 and K612 by GCN5L1 overexpression. Furthermore, GRP75‐K567/612 acetylation reduces the assemble of IP3R1‐GRP75‐VDAC complex, which in turn leads to the maintenance of ER calcium homeostasis and insulin sensitivity. Interestingly, during high‐fat diet feeding, mitochondria‐localized GCN5L1 is significantly translocated to the cytosol. This translocation attenuates the acetylation of GRP75 at K567/612 and consequently enhances ER‐mitochondrial calcium flux and induces ER stress. In parallel, deacetylation‐mimicking mutated GRP75‐K567/612 promotes IR in vivo. Consequently, these findings demonstrate that the acetylation‐dependent modification of GRP75 plays a functional role in regulating overnutrition‐induced IR.

## Graphical abstract

Overnutrition exacerbates insulin resistance (IR) and is linked to excessive mitochondrial protein acetylation. However, it remains unclear whether and how mitochondrial protein acetylation influences IR. GCN5L1‐dependent downregulation of GRP75 acetylation at K567/612 consequently enhances ER‐mitochondrial calcium flux and induces ER stress. The acetylation‐dependent modification of GRP75 plays a functional role in regulating overnutrition‐induced IR.

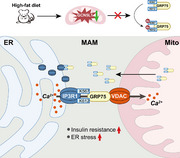

## 1 Introduction

Insulin resistance (IR), as a hallmark of type 2 diabetes (T2D), is an attractive target to ameliorate diabetes. Emerging evidence reveals diverse factors contributing to insulin resistance, including inflammatory signaling, endoplasmic reticulum (ER) stress, and mitochondrial dysfunction in peripheral tissues.^[^ ^1^, ^2^ ^]^ The liver, which plays a central role in maintaining whole body metabolic homeostasis, maintains an integrated mitochondrial‐ER network that communicates to regulate glucose homeostasis and insulin resistance.^[^ ^3^ ^]^ At the same time, the mechanism(s) underpinning the perturbation in mitochondrial and ER homeostasis in evoking IR remain to be fully elucidated.

Mitochondria, as the energy factory in cells, function as signaling transduction organelles communicating with the nucleus and other organelles to integrate metabolic signals to regulate cell function, fate, and homeostasis.^[^ ^4^ ^]^ Mitochondria and ER are physically connected, where a structure referred to as mitochondria‐associated ER membranes (MAMs), facilitates lipid, metabolites, and calcium transport to maintain ER and mitochondrial homeostasis.^[^ ^5^ ^]^ Several multiprotein tethering complexes maintain the MAM structure, including, MFN1/2, PACS2, Sig1R, and the IP3R‐GRP75‐VDAC complex.^[^ ^6^ ^]^ Studies have shown that the MAM structure, including the amount of ER‐mitochondrial contact and the distance between ER and mitochondria was closely related to high‐fat diet (HFD) induced hepatosteatosis and insulin resistance.^[^ ^7^, ^8^ ^]^ Interestingly, the tethering proteins display variant functions in the regulation of hepatic insulin sensitivity.^[^ ^9^, ^10^ ^]^ Among these tethering complexes, the IP3R‐GRP75‐VDAC complex has attracted broad attention in the investigation of disease development.^[^ ^11^ ^]^ It contributes to tethering ER and mitochondria as well as regulating ER to mitochondria calcium flux, which is a major ER‐mitochondrial communication signal regulating ER homeostasis and mitochondrial oxidative stress. Chronic disruption of MAM by deletion of the mitochondrial chaperone GRP75 impairs calcium transfer from ER to mitochondria and perturbs hepatic insulin signaling.^[^ ^12^, ^13^, ^14^ ^]^ In parallel, the induction of GRP75 has been shown to prevent HFD‐induced obesity and insulin resistance underlying the role of maintaining mitochondrial‐supercomplex stability.^[^ ^15^ ^]^ Surprisingly, IP3R1 deletion in the liver reduces mitochondrial calcium signals, which protects against HFD‐induced fatty liver but does not alter hepatic insulin signaling.^[^ ^7^, ^16^ ^]^ On the other hand, as a mitochondrial chaperone, GRP75 primarily localizes to the mitochondrial matrix and modulates stress responses through the mitochondrial unfolded protein response (UPR^MT^). The UPR^MT^ promotes insulin sensitivity.^[^ ^17^, ^18^ ^]^ Taken together, these findings suggest that fine‐tuning of MAM dynamics and putatively the IP3R‐GRP75‐VDAC complex in the regulation of hepatic insulin sensitivity may need further investigation.

Hepatic insulin resistance is a central feature underlying metabolic dysfunction‐associated fatty liver disease, and is associated with increased mitochondrial protein acetylation.^[^ ^19^ ^]^ Mechanistically, excess sugar or fat intake increases mitochondrial acetyl‐CoA levels, which in turn may facilitate mitochondrial protein acetylation with subsequent alterations in mitochondrial function and metabolism.^[^ ^19^, ^20^, ^21^ ^]^ Lysine acetylation is a dominant post‐translational modification in mitochondria in response to overnutrition, and feedback modulates enzyme activities for metabolic adaptation. Sirt3, the primary mitochondrial deacetylase, regulates fatty acid oxidation (FAO) and oxidative stress. However, hepatic Sirt3 does not influence insulin sensitivity.^[^ ^22^, ^23^, ^24^ ^]^ The adaptor protein GCN5L1/BLOC1S1 is a nutrient sensor in hepatocytes and is linked to the modulation of mitochondrial protein acetylation and in endo‐lysosome biology. Previously, we generated GCN5L1 liver knockout mice and found that GCN5L1 levels regulate several mitochondrial enzymes which modulate liver metabolic processes including glutaminolysis and gluconeogenesis.^[^ ^25^, ^26^ ^]^ Furthermore, these mice show resistance to nutrient‐induced fat accumulation in the liver.^[^ ^27^, ^28^ ^]^ Thus, we postulated that GCN5L1/BLOC1S1 knockout hepatocytes would be an intriguing model to explore the role of mitochondrial acetylation in insulin signaling and possibly to evaluate whether MAM homeostasis is similarly regulated by acetylation.

In this study, we found that hepatic deletion of GCN5L1 attenuated insulin signaling and that the mitochondrial localization of GCN5L1 sustained hepatic insulin signaling. By using immunoprecipitation‐mass spectrometry (IP‐MS) and mitochondrial protein acetylome analysis, we identified GRP75 acetylation as a target of GCN5L1. Mechanistically, we show that GRP75‐K567/612 acetylation modulates IP3R1‐GRP75‐VDAC assembly and in turn, regulates ER calcium homeostasis and hepatic insulin resistance. Overexpression of a deacetylated GRP75 mimic facilitated IR in vivo. Furthermore, we found HFD promoted hepatic mitochondrial GCN5L1 translocation to cytoplasm, which establishes a GCN5L1‐deleted environment in mitochondria to induce deacetylation of GRP75 on K567/612 thereby promoting hepatic IR. This modulation of GCN5L1 subcellular localization and its effect on GRP75 acetylation is a novel regulatory mechanism underlying overnutrition‐induced hepatic insulin resistance.

## 2 Results

### 2.1 Mitochondrial GCN5L1 Modulates Hepatic Insulin Signaling and ER Stress

**Figure 1:**
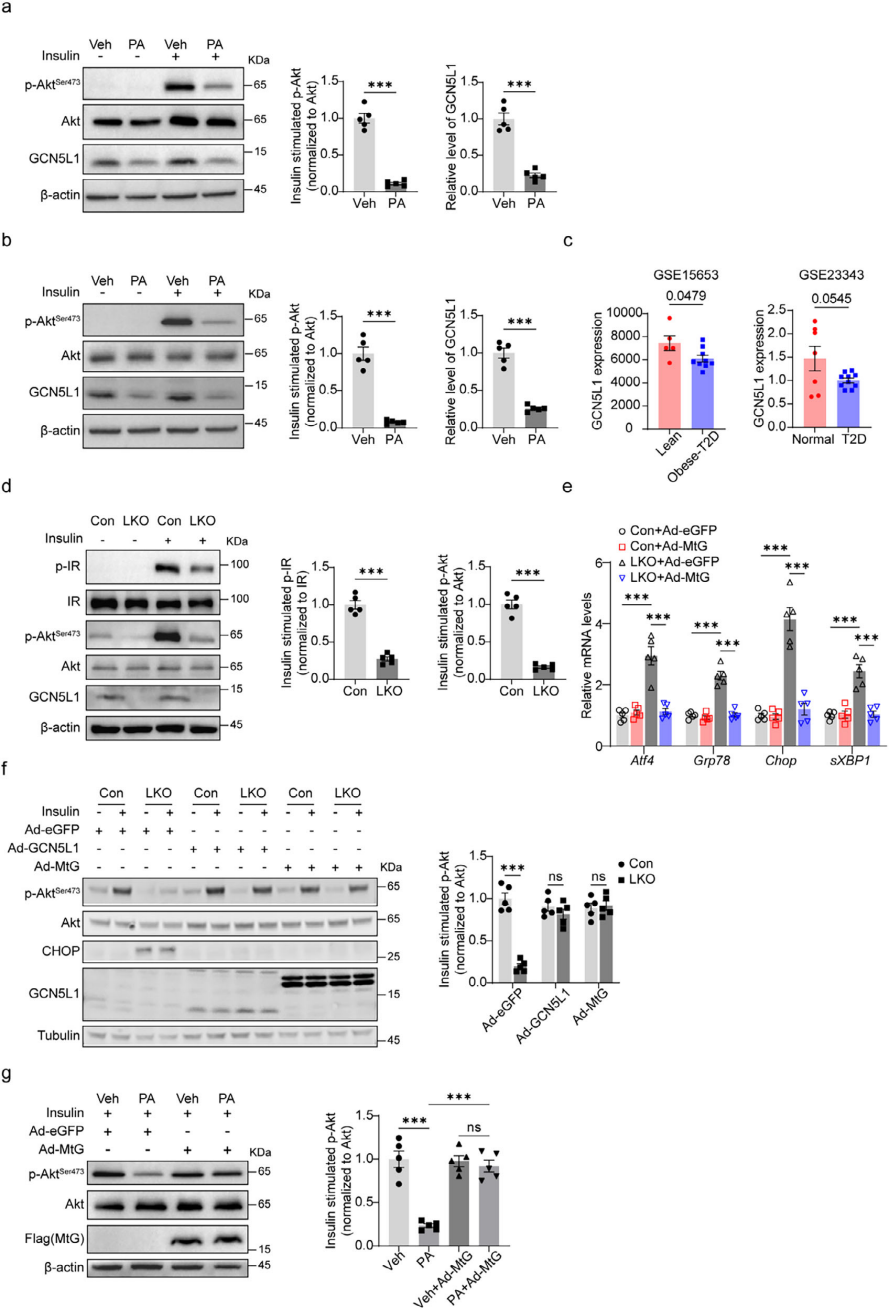
Mitochondrial GCN5L1 modulates hepatic insulin signaling and ER stress. a,b) Primary hepatocytes (a) from WT mice or HepG2 cells (b) were incubated with palmitate (PA: 0.75 mm, 18 h). Cell lysates were subjected to western blotting. Quantification of normalized ratios of p‐Akt or GCN5L1 upon PA treatment is shown. *n*  =  5. c) GCN5L1 transcriptional levels in liver biopsy specimens from T2D patients or healthy subjects from GSE 15 653 and GSE 23 343. d) Primary hepatocytes from Con (GCN5L1^flox/flox^) and LKO mice were incubated with or without insulin (10 nm, 15 min). Cell lysates were subjected to western blotting. Quantification of p‐Akt (S473)/Akt and p‐IR (Y1146)/IR upon insulin stimulation is shown. *n*  =  5. e) Primary hepatocytes were infected with adenovirus encoding mitochondrial restricted GCN5L1 (MtG) or eGFP control. Relative mRNA levels were measured by qPCR and normalized to control cells. *n*  =  5. f) Primary hepatocytes were infected with adenovirus encoding MtG, GCN5L1, or eGFP control and treated with insulin (10 nm, 15 min). Cell lysates were subjected to western blotting, and quantification of p‐Akt/Akt was normalized to control with insulin treatment. *n*  =  5. g) Primary hepatocytes from WT mice were infected with adenovirus encoding MtG, followed by treatment with palmitate (PA: 0.75 mm, 18 h) and insulin (10 nm, 15 min), cell lysates were subjected to western blotting, and quantification of p‐Akt/Akt was normalized to the treatment without PA or MtG. *n*  =  5. All values are expressed as means ± SEM. *P < 0.05, **P < 0.01, ***P < 0.001. Statistical analyses were performed using two‐tailed unpaired Student's *t‐*test (a–d), two‐way ANOVA with multiple comparisons (e,f), or one‐way ANOVA with multiple comparisons (g).

Prior to exploring the role of GCN5L1 in insulin signaling, we evaluated the correlation between GCN5L1 expression and insulin responsiveness. Here, incubation of primary murine hepatocytes and HepG2 cells with palmitate (PA) was employed to induce insulin resistance. Interestingly, palmitate reduced GCN5L1 expression along with inhibition of Akt phosphorylation upon insulin stimulation (**Figure** **1**a,b). Furthermore, we analyzed a transcriptional dataset of liver biopsy specimens from healthy subjects and patients with type 2 diabetes. Here, hepatic expression of GCN5L1 was decreased in insulin‐resistant patients (Figure 1c). Together, these data suggest that GCN5L1 expression negatively correlates with insulin resistance. To then directly determine whether GCN5L1 levels modulated hepatic insulin sensitivity, we assessed insulin signaling in primary hepatocytes from lean GCN5L1 LKO and control (Con, GCN5L1^flox/flox^) mice. In response to insulin stimulation, we observed the reduced phosphorylation of the insulin receptor (IR) on Y1146 and of Akt on S473 in GCN5L1 LKO hepatocytes (Figure 1d).

Mitochondrial dysfunction and oxidative stress are inextricably linked to the development of insulin resistance.^[^ ^29^ ^]^ We have previously shown that GCN5L1 deletion has no obvious effect on liver mitochondrial respiration.^[^ ^25^ ^]^ Similar mitochondrial respiration and comparable glycolysis rate were confirmed in primary hepatocytes from GCN5L1 LKO and Con mice (Figure S1a,b, Supporting Information). In addition, mitochondria are the hub of cellular reactive oxygen species (ROS) production, while excessive ROS attenuates insulin signaling in skeletal muscle and adipose tissue.^[^ ^30^ ^]^ GCN5L1 ablation accelerates ROS generation in primary hepatocytes and HepG2 cells.^[^ ^25^, ^31^ ^]^ We observed similar results in isolated liver mitochondria from GCN5L1 LKO mice (Figure S1c, Supporting Information). To evaluate whether excess mitochondrial ROS is responsible for IR in GCN5L1 LKO hepatocytes, we introduced mitochondrial electron transfer chain inhibitors to induce ROS and observed no difference in insulin‐stimulated p‐Akt levels in the presence of rotenone or antimycin (Figure S1d–f, Supporting Information). Furthermore, hepatocytes from GCN5L1 LKO and Con mice were incubated with the mitochondrial SOD mimetics Mito‐TEMPO followed by insulin stimulation. ROS scavenging had no effect on insulin‐induced Akt phosphorylation in LKO cells (Figure S1g, Supporting Information). These data suggest that GCN5L1 modulates hepatic IR through a mechanism distinct from perturbations in mitochondrial respiration or mitochondrial oxidative stress.

Given that the ER adjacent to mitochondria facilitates ER‐mitochondrial communication and insulin signaling, we examined whether GCN5L1 deletion affected ER homeostasis. Intriguingly, the transcriptional levels of ER stress markers were elevated in GCN5L1 LKO primary hepatocytes. Moreover, restoration of mitochondrial restricted GCN5L1 (MtG, GCN5L1 fused with mitochondrial targeting sequence from COX8)^[^ ^25^ ^]^ in LKO hepatocytes blunted the expression of this ER stress markers (Figure 1e). In parallel, the overexpression of GCN5L1 or the MtG construct similarly restored insulin‐mediated phosphorylation of Akt^S473^ in GCN5L1 LKO primary hepatocytes (Figure 1f). To validate this putative retrograde regulation of insulin signaling by MtG, we overexpressed MtG in primary hepatocytes from wild‐type mice in conjunction with PA‐induced IR and observed restoration of insulin sensitivity (Figure 1g). Together, these data suggest that loss of mitochondrial‐localized GCN5L1 may contribute to insulin resistance and imply that this process may be linked to the control of ER homeostasis.

To test whether ER stress mediates GCN5L1 LKO‐induced insulin resistance, ER stress inhibitors 4‐PBA and BiP inducer X were used to treat primary hepatocytes from GCN5L1 LKO and control mice. The expression of ER stress markers was restored by either 4‐PBA or BiP inducer X incubation (Figure S1h, Supporting Information). Meanwhile, these ER stress inhibitors restored insulin signaling in GCN5L1 LKO hepatocytes, indicating ER stress‐mediated GCN5L1 deletion induced IR (Figure S1i,j, Supporting Information). This further supports that insulin resistance caused by GCN5L1 deletion is closely associated with ER stress.

### 2.2 Identification of GRP75 as a Potential Mediator of GCN5L1 Ablation‐Induced Hepatic Insulin Resistance

**Figure 2:**
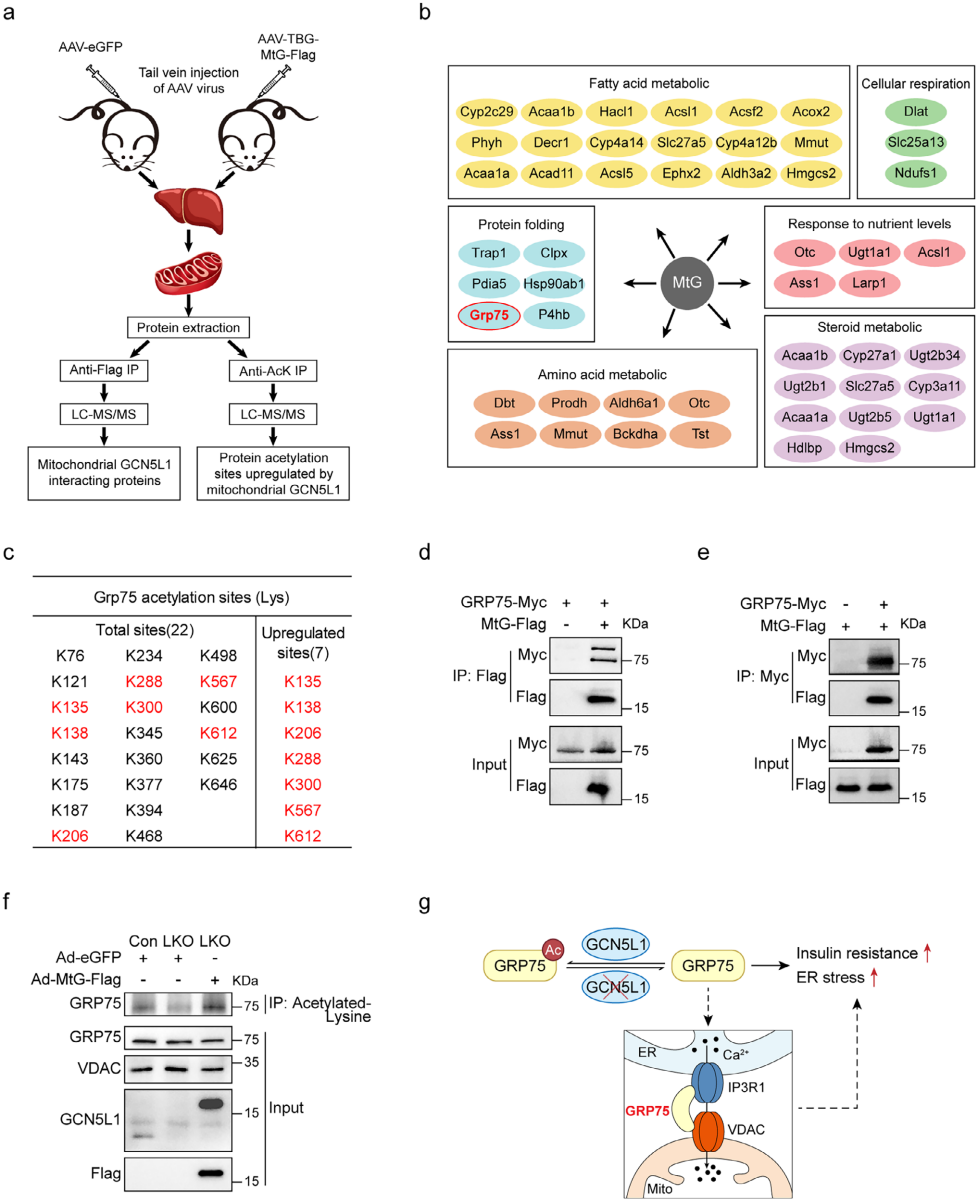
Identification of GRP75 as a potential mediator of GCN5L1 ablation‐induced hepatic insulin resistance. a) Illustration of strategies for proteomic analyses of interaction partners and acetylated proteins of mitochondrial GCN5L1. The crude mitochondrial fractions were isolated from AAV‐eGFP or AAV‐MtG‐flag livers, followed by accumulation of MtG‐flag for mass spectrometry to identify potential interaction partners. Meanwhile, crude mitochondrial proteins were trypsinized and immunoprecipitated by anti‐acetyl‐lysine antibody to accumulate acetyl‐peptides for mass spectrometry analysis. b) Identification of potential binding partners of mitochondrial GCN5L1 using IP‐MS was categorized based on the indicated Gene Ontology biological processes. c) Acetylated residues of GRP75 in murine livers under NCD were shown, and red font indicated significantly increased GRP75 residues in MtG expressing livers compared with Con mice. d,e) Co‐IP assay of GRP75 and GCN5L1, with MtG pulldown (D) or GRP75 pulldown (E). f) Levels of GRP75 acetylation in primary hepatocytes from control or GCN5L1 LKO mice with/without adeno‐MtG overexpression. g) Proposed mechanism of GCN5L1 LKO‐induced insulin resistance.

To investigate how mitochondrial restricted GCN5L1 (MtG) contributes to insulin signaling and ER homeostasis, we performed proteomic screenings to identify target(s) that both interact with GCN5L1 and show changes in protein acetylation. Two experimental strategies were employed. First, murine livers were transduced with adenovirus or adeno‐associated virus (AAV) carrying flag‐tagged MtG, followed by accumulation of MtG or eGFP control by flag resin or acetylated peptides by anti‐ACK antibody from crude liver mitochondrial fractions and analysis using mass spectrometry (MS) (**Figure** **2**a). Second, we purified His tagged murine GCN5L1 from *Escherichia coli* followed by incubation with liver mitochondrial extract and analysis with MS (Figure S2a, Supporting Information). We identified mitochondrial proteins as potential binding partners of GCN5L1. Surprisingly, both strategies highlighted the potential interaction of GRP75 with GCN5L1 (Figure 2b; Figure S2a, Supporting Information). In parallel, we concurrently analyzed the acetyl‐proteome. Here, over 100 peptides from 23 proteins showed significantly increased acetylation in the AAV‐MtG expressing livers compared with eGFP expression mice (Figure S2b, Table S1, Supporting Information). The subsequent integration of the IP‐MS and acetylome data again identified GRP75 and HMGCS2, a rate‐limiting enzyme in ketogenesis (Figure S2c, Table S2, Supporting Information). We focused on GRP75, given its link to insulin signaling. Surprisingly, GRP75 emerged as a highly acetylated mitochondrial protein with 22 acetylated residues in mouse livers. MtG induction specifically increased acetylation at 7 lysine residues (Figure 2c). Co‐immunoprecipitation (co‐IP) assays were then performed to validate the interaction of GRP75 and GCN5L1 (Figure 2d,e). Furthermore, to confirm that GCN5L1 regulates GRP75 acetylation, we pulled down acetylated proteins from GCN5L1 LKO and Con primary hepatocytes. We found that acetylated GRP75 levels decreased in GCN5L1‐KO hepatocytes but were restored by MtG overexpression (Figure 2f). Together, these data supported that mitochondrial GCN5L1 interacts with GRP75 and regulates its acetylation.

So far, we observed GCN5L1 deletion exacerbated ER stress signatures and blunted insulin signaling, and we identified GRP75 as a putative target to integrate these effects. GRP75 is predominantly located in the mitochondrial matrix and serves as a chaperone in mitochondria, responding to mitochondrial stress. The controversial functions of GRP75 ablation on insulin signaling have been reported to be associated with either mitochondrial integrated stress response (ISR) mediated UPR^MT^ to promote insulin sensitivity,^[^ ^32^ ^]^ or affected mitochondrial supercomplex turnover or MAM formation to induce IR.^[^ ^12^, ^15^ ^]^ These divergent findings suggest an unrevealed role of GRP75 in regulating insulin sensitivity. To evaluate GRP75 modulation in the regulation of insulin signaling, we generated HepG2 cells with GRP75 knockdown via siRNA transfection. We observed increased p‐Akt upon insulin stimulation in GRP75 knockdown cells (Figure S3a,b, Supporting Information), along with elevated ISR (Figure S3c, Supporting Information). This result implied that loss of GRP75 activates ISR but did not induce hepatic IR. On the other hand, as GRP75 is also a component of the ER‐mitochondrial calcium complex, we hypothesized that GCN5L1 might regulate GRP75 acetylation to modulate the ER‐mitochondrial calcium complex activity, thereby affecting ER homeostasis and insulin sensitivity (Figure 2g).

### 2.3 Mitochondrial GCN5L1 Regulates ER‐Mitochondrial Contact and Calcium Flux

**Figure 3:**
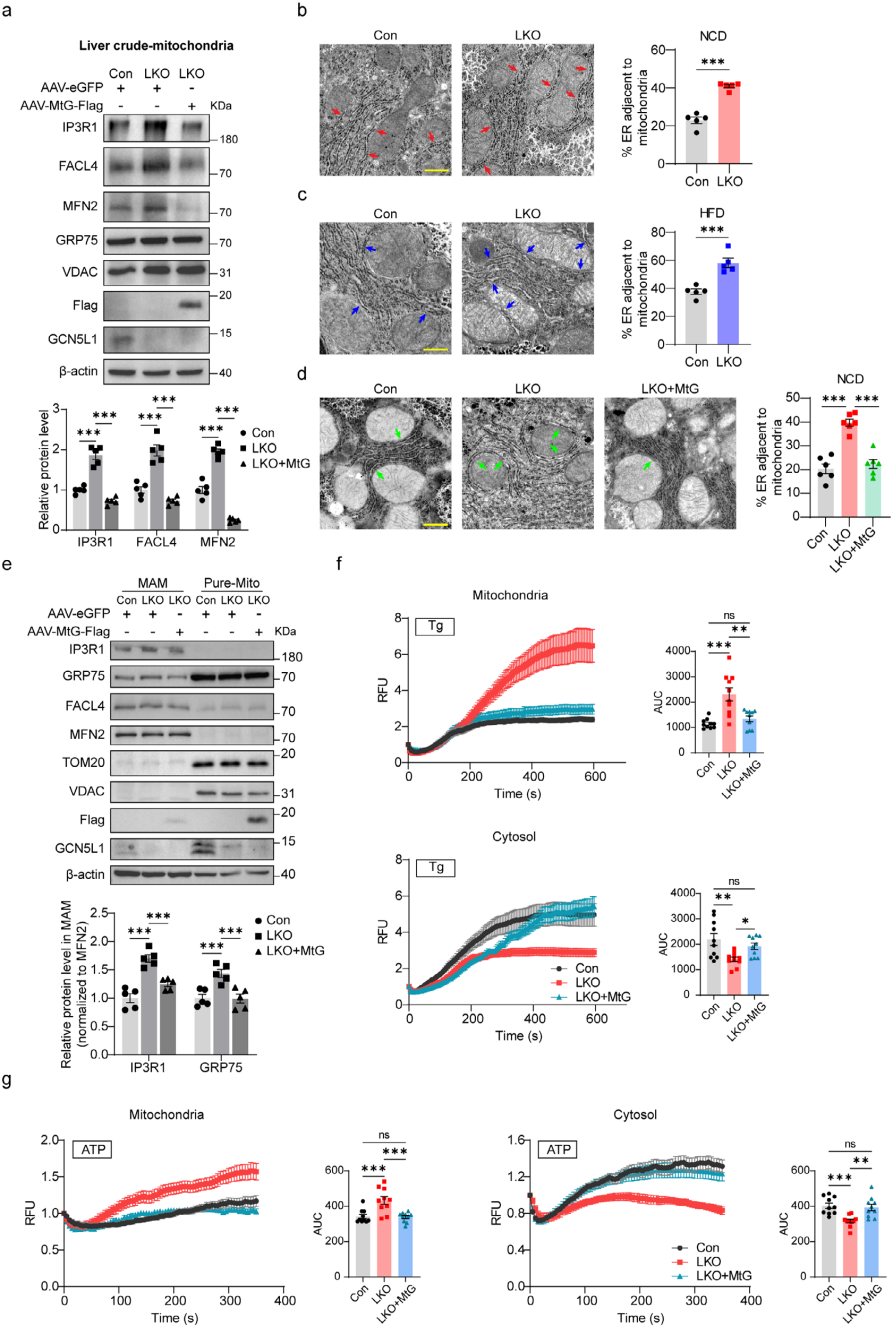
Mitochondrial GCN5L1 regulates ER‐mitochondrial contact and calcium flux. a) Western blot analysis of crude mitochondrial fractions from Con, GCN5L1 LKO, and LKO with AAV‐MtG expression livers. IP3R1, FACL4, and MFN2 levels were normalized to β‐actin. *n*  =  5. b,c) TEM images of GCN5L1 LKO and control livers from NCD (b) and HFD for 16 weeks (c). ER‐mitochondrial contact index was calculated under each condition. Scale bar, 5 µm. Mitochondria and ER were delimited using the freehand selection tool of lmageJ. Quantitation of ER length adjacent to mitochondria normalized by total ER length and by mitochondrial perimeter. MAM structures were indicated with arrows. d) TEM images of control, GCN5L1 LKO, and LKO with AAV‐MtG expression livers upon NCD feeding. Scale bar, 5 µm. Mitochondria and ER were delimited using the free hand selection tool of lmageJ. Quantitation of ER length adjacent to mitochondria normalized by total ER length and by mitochondrial perimeter. MAM structures were indicated with arrows. e) Pure mitochondria (Pure‐Mito) and MAM fractions were purified from mouse livers using ultracentrifuge and subjected to immunoblotting. IP3R1 levels were normalized to MFN2, MAM marker. *n*  =  5. (f,g) Calcium images from primary hepatocytes of control, GCN5L1 LKO, and LKO with adeno‐MtG overexpression were recorded upon Thapsigargin (Tg, 1 µm) (f) or ATP (10 µm) (g) stimulation. Mitochondrial calcium signal was indicated by pCAG mito‐RCaMP1h (*n * =  10), and cytosolic calcium signal was indicated by pGP‐CMV‐jGCaMP7f (*n * =  10). AUC, Area Under the Curve. All values are expressed as means ± SEM. *P < 0.05, ***P < 0.001. Statistical analyses were performed using two‐tailed unpaired Student's *t‐*test (b,c), two‐way ANOVA with multiple comparisons (a,e), or one‐way ANOVA with multiple comparisons (d,f,g).

To assess the structural interactions in this biology, we evaluated the extent of ER‐mitochondrial contact as well as IP3R‐GRP75‐VDAC complex formation comparing Con and LKO mice. Crude mitochondrial fractions were isolated and showed increased FACL4 and MFN2, which are MAM markers^[^ ^33^ ^]^ as well as IP3R1 levels in LKO crude mitochondria, in addition, MtG expression in LKO livers restored these protein expression (**Figure** **3**a). These findings suggest increased ER‐mitochondrial calcium complex and MAM formation in LKO livers. To confirm this result, we used transmission electron microscopy (TEM) and observed more MAM structure in both normal chow diet (NCD) and HFD GCN5L1 LKO livers compared to controls (Figure 3b,c). Again, the increased MAM structure in GCN5L1 LKO livers was restored by MtG expression (Figure 3d). To verify whether accumulated IP3R1 was accompanied by MAM formation, we purified MAM from livers and found that IP3R1 was increased in MAM of LKO mice, suggesting that accumulation of IP3R1 in crude mitochondria was associated with increased MAM formation in GCN5L1 LKO livers (Figure 3e). These data revealed that IP3R1 was recruited in the MAM of GCN5L1 LKO livers.

To evaluate the function of IP3R1 enrichment in MAMs of GCN5L1 LKO livers, we measured cytosolic and mitochondrial calcium levels upon Thapsigargin discharge and ATP stimulation. GCN5L1 loss significantly enhanced mitochondrial calcium levels, while restoration of MtG restored cytosolic and mitochondrial calcium levels in GCN5L1‐depleted hepatocytes (Figure 3f,g). Together, these data imply that IP3R1 enrichment in MAMs of GCN5L1 LKO hepatocytes enhances ER‐mitochondrial calcium flux.

### 2.4 GCN5L1 Deletion Reduces Insulin Signaling in Primary Hepatocytes through Disturbance of ER‐Mitochondrial Calcium Homeostasis

**Figure 4:**
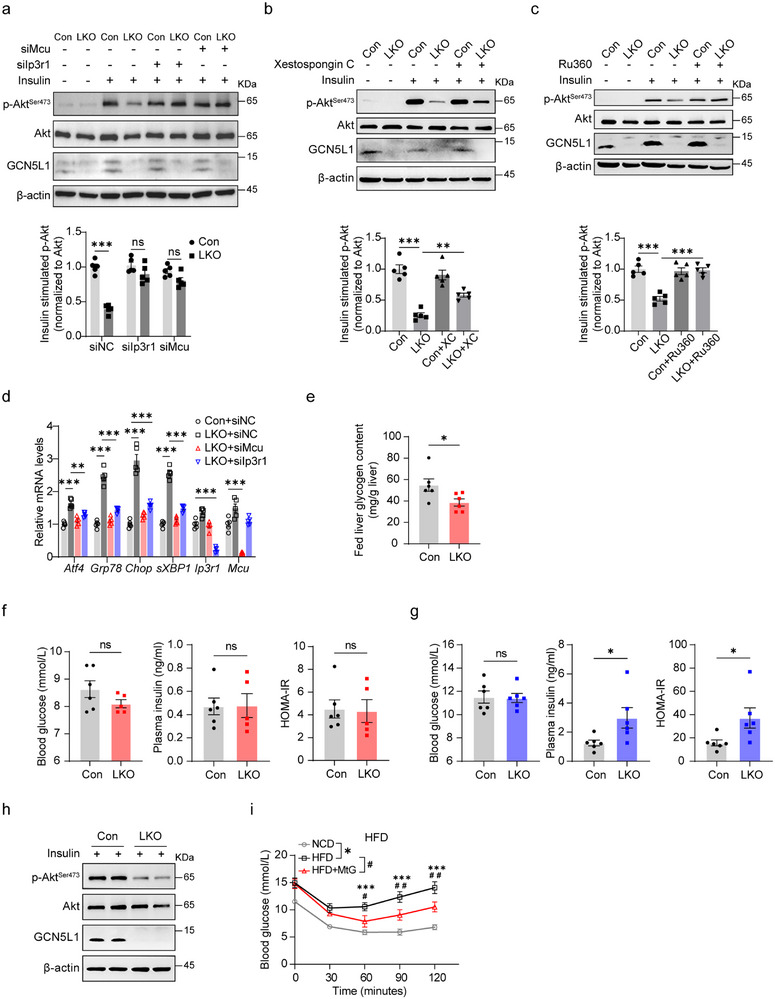
GCN5L1 deletion reduces insulin signaling in primary hepatocytes through disturbance of ER‐mitochondrial calcium homeostasis. a) Primary hepatocytes from GCN5L1 LKO and Con mice were incubated with siRNA to knock down IP3R1 or MCU, followed by incubation with insulin for 15 min. P‐Akt/Akt levels were analyzed by immunoblotting and normalized to Con with insulin treatment. *n * =  5. b) Hepatocytes were isolated from GCN5L1 LKO and Con mice, and incubated with IP3R1 inhibitor XC (1 µm) for 18 h, followed by incubation with insulin for 15 min. P‐Akt levels were analyzed by immunoblotting and normalized to control with insulin treatment. *n*  =  5. c) Hepatocytes from GCN5L1 LKO and Con mice were incubated with MCU inhibitor Ru360 (3 µm) for 18 h, followed by incubation with insulin for 15 min. P‐Akt levels were analyzed by immunoblotting and normalized to control with insulin treatment. *n* = 5. d) Primary hepatocytes from GCN5L1 LKO and Con mice were incubated with siRNA to knock down IP3R1 or MCU, relative mRNA levels were measured by qPCR and normalized to Con cells. *n* = 5. e) Liver glycogen contents were measured in 24‐week NCD‐fed mice. Con, *n *= 6; LKO, *n *= 6. f) Blood glucose and plasma insulin levels were measured in NCD‐fed mice. HOMA‐IR was calculated. Con, *n *= 6; LKO, *n *= 5. g) Blood glucose and plasma insulin levels were measured in mice with HFD for 16 weeks. HOMA‐IR was calculated. Con, *n *= 6; LKO, *n* = 6. h) Mice fed with HFD for 16 weeks were i.p. injected with insulin at 0.75 U kg^−1^ body weight, and liver tissues were collected for immunoblotting analysis. i) Insulin tolerance test (ITT) was performed in 8‐week HFD‐fed mice with AAV‐eGFP or AAV‐MtG expression and age‐matched NCD‐fed mice. *n *= 7. All values are expressed as means ± SEM. *P < 0.05, **P < 0.01, ***P < 0.001. ns, not significant. Statistical analyses were performed using two‐way ANOVA with multiple comparisons (a,b), two‐tailed unpaired Student's *t*‐test (e–g), or one‐way ANOVA with multiple comparisons (c,d,i).

To validate the role of ER‐mitochondrial calcium disorder in GCN5L1 loss‐induced hepatic insulin resistance, we introduced siRNA to modulate the expression of IP3R1 in hepatocytes. IP3R1 knockdown showed no effect on Akt^S473^ phosphorylation in control cells but restored insulin‐stimulated p‐AktS473 levels in GCN5L1‐deficient hepatocytes (**Figure** **4**a; Figure S4a,b, Supporting Information). In parallel, we incubated primary hepatocytes with Xestospongin C (XC), an IP3R1 inhibitor.^[^ ^12^ ^]^ Similar results were observed (Figure 4b). As mitochondrial calcium import is regulated in part by the mitochondrial calcium uniporter (MCU),^[^ ^34^ ^]^ we then knocked down MCU by siRNA or inhibited calcium uptake by using the MCU inhibitor Ru360 and found that Akt^S473^ phosphorylation was restored in GCN5L1 LKO (Figure 4a,c; Figure S4c,d, Supporting Information). In addition, the transcriptional levels of ER stress markers were restored by either knockdown of IP3R1 or MCU in GCN5L1 LKO primary hepatocytes (Figure 4d). Together, these data imply that IP3R1 enrichment in MAMs of GCN5L1 LKO hepatocytes enhances ER‐mitochondrial calcium flux, and concurrently promotes insulin resistance.

The experimental readouts of hepatocellular insulin action are insulin‐stimulated glycogen synthesis and insulin‐regulated transcripts. To assess the function of GCN5L1 on insulin action, we first isolated primary hepatocytes from Con and LKO mice, followed by incubation with an appropriate amount of insulin to induce glycogen synthesis or related gene expression (Figure S5a, Supporting Information). As expected, insulin significantly increased lipid synthesis‐related gene expression in control hepatocytes; conversely, LKO hepatocytes were unresponsive to that (Figure S5b, Supporting Information). Inhibition of IP3R1 with XC significantly restored insulin‐stimulated gene expression in LKO hepatocytes (Figure S5b, Supporting Information). We then examined glycogen synthesis upon insulin stimulation in primary hepatocytes. Primary hepatocytes were isolated, followed by incubation with low glucose DMEM mimicking a fasting state or high glucose DMEM plus insulin mimicking the fed status. High glucose media plus insulin robustly stimulated glycogen synthesis in control but not LKO hepatocytes, while IP3R1 inhibition again rescued the impaired glycogen synthetic ability in LKO hepatocytes (Figure S5c, Supporting Information).

To examine insulin action in vivo, we measured hepatic glycogen contents under fed conditions in mice on a normal chow diet (NCD). In parallel with the hepatocyte studies, GCN5L1 LKO hepatic glycogen was significantly decreased in LKO livers without effect on plasma glucose and insulin levels on the HOMA‐IR calculation (Figure 4e,f). In contrast, on a high‐fat diet (HFD), LKO mice exhibited elevated plasma insulin levels and a higher HOMA‐IR, with the maintenance of blood glucose levels indicating the development of insulin resistance (Figure 4g). To verify this, we measured insulin signaling in mouse livers and found that in vivo insulin stimulated Akt phosphorylation dramatically decreased in GCN5L1 LKO compared with control mice (Figure 4h). MtG expression was observed to restore insulin signaling in primary hepatocytes upon PA‐induced IR in Figure 1g, we then assessed the in vivo effect of MtG expression in HFD induced IR. HFD fed wildtype mice displayed significant IR compared to NCD‐fed mice, while MtG overexpression significantly enhanced insulin sensitivity in vivo (Figure 4i). Together, these data further support the notion that the depletion of GCN5L1 impairs insulin action in primary hepatocytes and livers, suppressing glycogen and lipid synthesis. This occurs, in part, due to GCN5L1‐mediated disruption of the activation of the ER‐mitochondrial calcium complex.

### 2.5 K567/612 Acetylation of GRP75 Modulates IP3R1‐GRP75‐VDAC Complex Assembly

**Figure 5:**
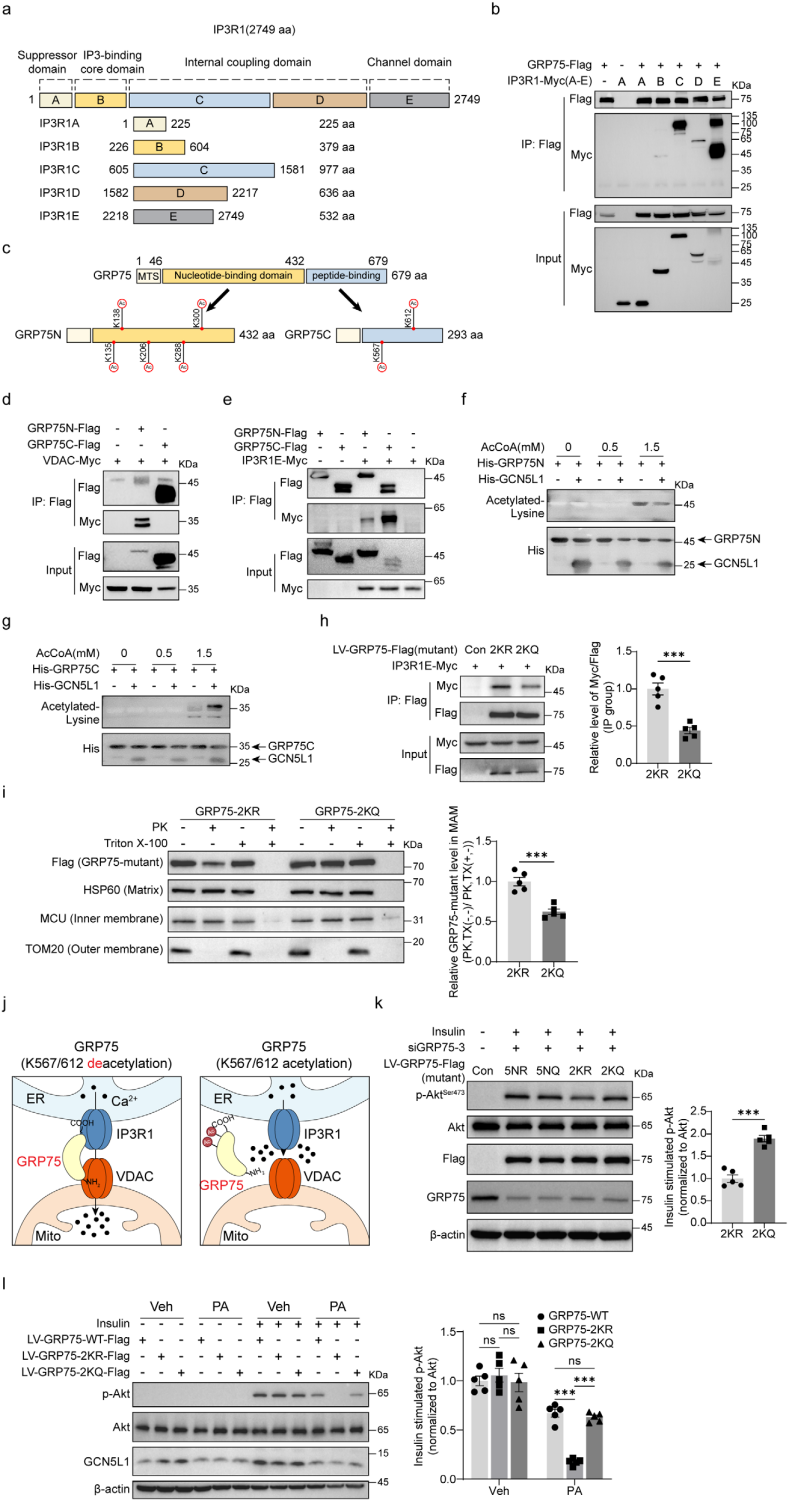
K567/612 acetylation of GRP75 modulates IP3R1‐GRP75‐VDAC complex assembly. a) Illustration indicated domains and fragments of IP3R1. b) Co‐IP analysis of the interaction of GRP75 and IP3R1 fragments. c) Illustration of GRP75 domains. The GRP75C fragment was generated by the fusion of the GRP75C domain with the MTS (mitochondrial targeting sequence) of GRP75. d) Co‐IP analyzed the interaction of VDAC with GRP75 domains. e) Co‐IP analyzed the interaction of IP3R1 with GRP75 domains. f,g) In vitro acetylation assay. GRP75N (f) or GRP75C (g) domains were purified from BL21 and incubated with indicated doses of acetyl‐CoA and purified GCN5L1. Reactions were subjected to western blotting to analyze GRP75 acetylation. h) Co‐IP assay analyzed the interaction of GRP75‐2KR or 2KQ with IP3R1. Quantification of IP3R1 interaction with GRP75 mutants was calculated by normalization of IP3R1E to flag‐GPR75 mutants in immunoprecipitation samples. *n* = 5. i) Proteinase K protection assay was performed in crude mitochondrial fractions from AAV‐GRP75‐2KR or 2KQ expression livers to determine the localization of GRP75‐2KR and 2KQ. HSP60, MCU, and TOM20 were used to indicate the inner or outer of mitochondrial fractions. Relative GRP75‐mutant (flag) levels in MAM were normalized. Levels of GRP75‐mutant on MAM are representative of the Flag ratios of the PK, TX (−, −) to PK, TX (+, −) groups. *n* = 5. j) Illustration proposed a pattern of GRP75 acetylation on K567/612 in the regulation of IP3R1‐GRP75‐VDAC complex. k) GRP75‐2KR/2KQ (K567/612 modification) and GRP75‐5NR/5NQ (K135/138/206/288/300 modification) were transduced into HepG2 cells with knockdown of endogenous GRP75, p‐Akt levels were analyzed upon insulin stimulation. *n* = 5. l) HepG2 cells were infected with lentivirus expression or GRP75‐2KR/2KQ or WT, followed by incubation with/without PA for 18 h and insulin stimulation. Cell lysates were analyzed by immunoblotting. P‐Akt/Akt levels were analyzed by immunoblotting and normalized to Con with insulin treatment. *n* = 5. All values are expressed as means ± SEM. *P < 0.05, ***P < 0.001. ns, not significant. Statistical analyses were performed using two‐tailed unpaired Student's *t*‐test (h,i,k), or two‐way ANOVA with multiple comparisons (l).

Thus far, we have shown that GCN5L1 deletion is accompanied by the recruitment of IP3R1 to MAMs, and that GCN5L1 interacts with, and regulates GRP75 acetylation. Putting this together, we hypothesized that GRP75 acetylation may alter its interaction with IP3R1 and MAM complex assembly. To test this, we first interrogated this complex assembly. As background, IP3R1 is an ER transmembrane protein with a molecular weight over 300 kDa. Utilizing 5 distinct IP3R1 coupling domain fragments^[^ ^35^ ^]^ (**Figure** **5**a), we first evaluated the interaction of GRP75 with these different fragments. We found that GRP75 had a strong affinity for fragment C and channel fragment E (Figure 5b). As previously identified and illustrated in Figure 2c, GCN5L1 is associated with the acetylation of 7 lysine residues of GRP75. To examine which acetylation residue(s) of GRP75 contribute to IP3R‐GRP75‐VDAC complex assembly, we generated GRP75 fragments encompassing the N terminal (GRP75N) and the C terminal (GRP75C) as shown (Figure 5c). We then investigated the interaction of these GRP75 fragments with VDAC and IP3R1. We introduced a co‐IP assay to verify the complex assembly and found that GRP75N had a higher affinity for VDAC while GRP75C preferentially bound to IP3R1 (Figure 5d,e). Together, these data suggest that GRP75 functions as a “bridge” to connect IP3R1 and VDAC. As 5 out of 7 acetylated residues were on the GRP75N part and 2 acetylated residues on the GRP75C part, we employed an in vitro assay to evaluate whether GCN5L1 mediated acetyl‐modification of GRP75 modified these interactions. Mitochondrial acetylation appears to be driven mostly via a non‐enzymatic manner and an alkaline environment.^[^ ^36^ ^]^ We incubated purified GRP75 truncated proteins in an alkaline buffer plus acetyl‐CoA with/without GCN5L1 to mimic the mitochondrial matrix environment. We found that GCN5L1 promoted acetylation on GRP75C but not GRP75N (Figure 5f,g). We then asked whether GRP75 acetylation on K567/612 affects complex assembly. To further explore this, we generated GRP75‐2KR mutation, which substituted K567 and K612 with arginine to mimic GRP75 deacetylation, and these same residues with glutamine to mimic K567/612 acetylation (Figure S6a, Supporting Information). The co‐IP assay indicated that deacetylation of GRP75 on K567/612 increased affinity for IP3R1 (Figure 5h). We then posed another question: whether GRP75 deacetylation impacts its MAM localization. To assess this, a Proteinase K protection assay was performed. Crude mitochondrial fractions were isolated from AAV‐GRP75‐2KR/2KQ expression livers and subsequently incubated with Proteinase K and a detergent reagent. Since the mitochondrial membrane shields mitochondrial proteins from Proteinase K digestion, in the absence of detergent, the MAM proteins would be digested by Proteinase K. Proteinase K partially reduced GRP75‐2KR levels but had no effect on 2KQ, indicating that GRP75‐K567/612 deacetylation mimics exhibited increased localization in MAMs (Figure 5i). These data further support that GRP75 acts as a bridge to connect IP3R1 and VDAC in MAM, and that the deacetylation of GRP75 on K567/612 enhances the interaction between GRP75 and IP3R1, to facilitate the formation of the ER‐mitochondrial calcium complex (schematized in Figure 5j).

To confirm that K567/612 acetylation rather than other lysine acetylation on GRP75, regulated IR, we replaced endogenous GRP75 with mutants and showed that K567/612 deacetylation (2KR) mutations decreased p‐AKT levels compared with 2KQ, while K135/138/206/288/300 acetylation of GRP75 had no effect on insulin sensitivity (Figure 5k). We overexpressed GRP75‐WT and K567/612 mutations in HepG2 cells and found 2KR or 2KQ mutations had no effect on insulin‐stimulated p‐AKT compared to GRP75‐WT, interestingly 2KR mutation worsened PA‐induced IR in HepG2 cells compared with the WT or 2KQ mutation (Figure 5l). To further validate the regulatory role of GCN5L1 and GRP75‐K567/612 acetylation in the IP3R1‐GRP75‐VDAC complex, we performed Blue‐native PAGE with primary hepatocytes from GCN5L1 LKO and control mice, as well as GRP75‐2KR and GRP75‐2KQ mice. As IP3R1 forms a tetramer in its active state,^[^ ^19^ ^]^ the molecular weight of the IP3R1‐GRP75‐VDAC complex is predicted to be ≈880 kDa. All components of the complex were identified via immunoblotting using individual‐specific antibodies; the complex was significantly increased in GCN5L1 LKO hepatocytes compared to controls (Figure S6b, Supporting Information). Similarly, GRP75‐2KR‐overexpressing hepatocytes exhibited increased complex levels compared to the GRP75‐2KQ group (Figure S6c, Supporting Information). In conclusion, our data support that the acetylation of GRP75 on K567/612 modulates IP3R1‐GRP75‐VDAC complex assembly to regulate insulin signaling.

### 2.6 GCN5L1/GRP75 Axis is Critical for Overnutrition‐Induced IR via Modulating ER‐Mitochondria Calcium Homeostasis

**Figure 6:**
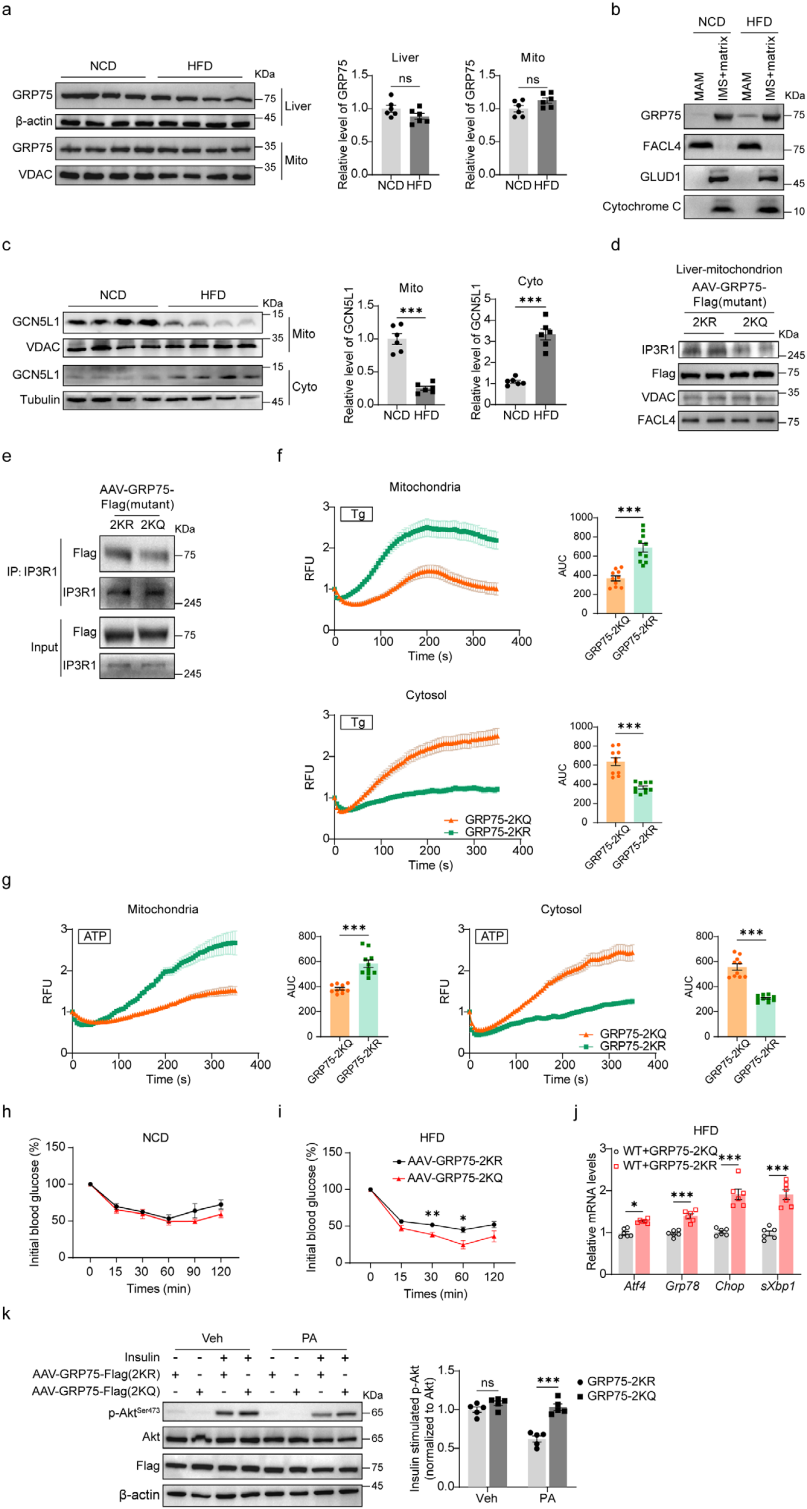
GCN5L1/GRP75 axis is critical for overnutrition‐induced IR via modulating ER‐mitochondria calcium homeostasis. a) Liver and mitochondrial lysates from NCD and HFD for 16 weeks fed wildtype mice were analyzed by western blotting. Quantifications of GRP75 expression are shown. *n* = 6. b) MAM and mitochondrial proteins were separated from NCD and HFD for 16 weeks livers from wildtype mice and subjected to western blotting. c) GCN5L1 protein levels were analyzed in crude mitochondrial and cytosolic fractions from NCD and 16‐week‐HFD livers from wild‐type mice. Relative GCN5L1 protein levels in HFD were normalized to related NCD. *n* = 6. d) Crude mitochondrial fractions from AAV‐GRP75‐2KR/2KQ expression livers under NCD were subjected for western blotting analysis of IP3R1. e) Interaction of endogenous IP3R1 with GRP75‐2KQ or 2KR in livers from (d). f,g) AAV‐GRP75‐2KR/2KQ were expressed in wildtype mouse livers through tail‐vein injection. Primary hepatocytes were isolated for calcium imaging with Tg (f) stimulation or ATP (g) stimulation. Mitochondrial calcium signal was indicated by pCAG mito‐RCaMP1h (*n *= 10), and cytosolic calcium signal was indicated by pGP‐CMV‐jGCaMP7f (*n *= 10). AUC, Area Under the Curve. h,i) AAV‐GRP75‐2KR/2KQ were expressed in mouse livers. ITTs were performed 3 weeks after injection (h) or an additional 2 weeks of HFD feeding (i). *n *= 6 mice per group. j) Wildtype mice were injected with AAV‐GRP75‐2KR/2KQ, followed by 2‐week HFD feeding. Relative mRNA levels were measured by qPCR and normalized to GRP75‐2KQ. *n *= 6. k) Primary hepatocytes were isolated from AAV‐GRP75‐2KQ/2KR mice and incubated with/without PA, followed by insulin stimulation and immunoblotting analysis. P‐Akt/Akt levels were analyzed and normalized to GRP75‐2KR with insulin and vehicle treatment. *n* = 5. All values are expressed as means ± SEM. *P < 0.05, **P < 0.01, ***P   0.001. ns, not significant. Statistical analyses were performed using two‐tailed unpaired Student's *t*‐test (a,c,i), one‐way ANOVA with multiple comparisons (f,g), or two‐way ANOVA with multiple comparisons (j,k).

Given that overnutrition disrupts insulin signaling, we measured GRP75 expression in HFD‐induced insulin‐resistant. We found that GRP75 protein levels were comparable in HFD and NCD‐fed murine livers as well as mitochondrial fractions (**Figure** **6**a). Interestingly, the location of GRP75 on the MAM was significantly increased in IR livers (Figure 6b) and hepatocytes (Figure S7a, Supporting Information), suggesting that HFD promoted GRP75 translocation associated with IR. In parallel with GRP75 translocation, HFD reduced mitochondrial localization of GCN5L1 but enhanced GCN5L1 localization to the cytosol (Figure 6c). A similar result was found in PA‐treated primary hepatocytes and HepG2 cells (Figure S7b,c, Supporting Information), suggesting that the decreased mitochondrial localization of GCN5L1 may enable this disruption in insulin signaling. The mitochondrial extrusion of GCN5L1 may result in the deacetylation of GRP75 on K567/612, which may consequently induce IP3R1‐GRP75‐VDAC complex assembly. To test this, we generated AAV‐GRP75‐2KQ and AAV‐GRP75‐2KR expression vectors and injected them through the tail veins of C57BL/6 mice. Hepatic expression of GRP75‐2KQ and GRP75‐2KR exhibited comparable basic mitochondrial function, including OXPHOS expression, mitochondrial DNA copy number, ROS generation, and mitochondrial biogenesis‐related gene expression (Figure S7d–g, Supporting Information). We found that GRP75‐2KR recruited more IP3R1 protein in crude liver mitochondrial fractions compared to GRP75‐2KQ mutation (Figure 6d). In parallel, GRP75‐2KR increased affinity with IP3R1 compared to GRP75‐2KQ (Figure 6e). We then evaluated the function of GRP75‐K567/612 mutants on the regulation of ER‐mitochondrial calcium flux. Primary hepatocytes were isolated from AAV‐GRP75‐2KQ and AAV‐GRP75‐2KR expression mice, the cytosolic and mitochondrial calcium levels were monitored upon Thapsigargin or ATP stimulation. GRP75‐2KR expression significantly enhanced mitochondrial calcium levels but reduced cytosolic calcium levels compared to GRP75‐2KQ expression, suggesting that GRP75‐K567/612 deacetylation promoted its affinity to IP3R1 to induce ER‐mitochondrial calcium flux (Figure 6f,g). Furthermore, GRP75‐2KR and GRP75‐2KQ mice displayed comparable insulin sensitivity under normal chow (Figure 6h). Intriguingly, GRP75‐2KR mice showed significant insulin resistance compared to GRP75‐2KQ mice with comparable body weight after 2 weeks on HFD (Figure 6i). Meanwhile, ER stress‐related gene expression was increased in GRP75‐2KR mice with 2‐week HFD compared to GRP75‐2KQ mice (Figure 6j), suggesting GRP75‐K567/612 deacetylation results in ER stress and IR in vivo. Moreover, primary hepatocytes from GRP75‐2KR mice exhibited modest blunting of insulin signaling, which was further augmented upon PA treatment (Figure 6k). These data further underscore the role of K567/612 acetylation of GRP75 in the regulation of IR, while simultaneously demonstrating that HFD‐induced loss of mitochondrial GCN5L1 enables GRP75 deacetylation on K567/612 during the development of hepatic IR.

Collectively this study finds that GRP75 acetylation is crucial to regulating the assembly of the calcium complex and insulin sensitivity. HFD‐induced loss of mitochondria and the exclusion of GCN5L1 from mitochondria enable GRP75‐K567/612 deacetylation, which promotes IP3R1‐GRP75‐VDAC complex assembly to further induce ER stress and insulin resistance.

## 3 Discussion

In the current study, we aimed to explore mitochondrial protein acetylation in the regulation of hepatic insulin resistance, which could be a fundamental mechanism of overnutrition‐induced insulin resistance. Acetylation is the predominant post‐translational modification in mitochondria, which connects nutrient sensing with metabolic adaptation. Dysregulation of mitochondrial protein acetylation, for instance in Sirt3 knockout, induces hepatic lipid disorder.^[^ ^37^ ^]^ Lipid accumulation is the major risk factor for hepatic insulin resistance, underlying the mechanism of increased inflammation, the diacylglycerols (DAG) destroyed pathway decay, and ER stress.^[^ ^38^ ^]^ However, whether acetyl‐modification is directly involved in the development of insulin resistance remains unresolved. At the same time, although Sirt3 is the major mitochondrial deacetylase, its gene modification does not exhibit a regulatory function on hepatic insulin resistance, in NCD or HFD feeding conditions.^[^ ^22^, ^24^ ^]^ Thus, we generated the GCN5L1 liver knockout mouse, which has been shown to modulate both mitochondrial protein acetylation and activity of enzymes involving in glutaminolysis, fatty acid oxidation,^[^ ^26^, ^31^ ^]^ and lysosomal lipolysis.^[^ ^28^ ^]^ In the current study, we utilized protein interactome and acetylome to identify potential targets of GCN5L1 in liver mitochondria, which consequently affect insulin signaling in GCN5L1‐deleted primary hepatocytes. We identified GRP75‐K567/612 deacetylation is associated with GCN5L1 deletion‐induced insulin resistance.

Our proteomic data identified the potential substrates of mitochondrial GCN5L1 including Hadha, which has been reported to regulate FAO.^[^ ^27^ ^]^ The acetyl‐proteomic data demonstrated highly acetylated liver mitochondrial proteins, such as Cps1, Glud1, Hmgcs2, and Got2, which are consistent with existing evidence.^[^ ^39^, ^40^, ^41^, ^42^ ^]^ Interestingly, we demonstrated GRP75 as a protein with multiple acetylation residues and with a high affinity for binding to GCN5L1 (Figure 2). Together, this led us to consider GRP75 as a putative mediator in GCN5L1 depletion induced IR. Especially given that the IP3R‐GRP75‐VDAC complex is recognized to maintain MAM structure and is thought to contribute toward the development of IR.^[^ ^11^ ^]^ Previous studies exploited cell models to modulate GRP75 expression and demonstrate the function of GRP75 on the regulation of MAM integrity and insulin sensitivity.^[^ ^12^, ^43^, ^44^ ^]^ These research works demonstrate that GRP75 deficiency inhibits insulin signaling via different mechanisms. Tubbs et al. report GRP75 knockdown in hepatic cell lines with shRNA impairs MAM integrity to reduce insulin signaling.^[^ ^12^ ^]^ Interestingly, Zhao et al. show evidence that GRP75 knockdown minimally affects MAM integrity but blocks insulin signaling by regulating mitochondrial‐supercomplex turnover.^[^ ^15^ ^]^ Both works utilize shRNA‐based GRP75 knockdown stable cell lines for investigation. We observed GRP75 with siRNA significantly enhanced insulin signaling in primary hepatocytes, suggesting that short‐term knockdown of GRP75 might activate different mitochondrial stress responses rather than long‐term knockdown. In addition, as homozygous knockout of GRP75 is embryonic lethal,^[^ ^45^ ^]^ and tissue‐specific knockout of GRP75 affects organ development,^[^ ^46^ ^]^ this model is limited to explore the in vivo function of GRP75 with respect to MAM homeostasis and in IR development. Here, we demonstrate that mitochondrial acetylation regulator GCN5L1 is crucial for maintaining hepatic insulin sensitivity. Mitochondrial restricted GCN5L1 reverses palmitate‐induced IR. GCN5L1 binds to GRP75 and modulates its acetylation on K567/612, which is critical for ER‐mitochondrial calcium homeostasis and insulin sensitivity. GRP75‐2KR mutation mimics K567/612 deacetylation is crucial for IP3R1 recruitment to MAM to induce ER stress and IR. It is interesting that the GRP75‐2KR did not have a profound effect on insulin signaling under normal chow or in the absence of PA in primary hepatocytes (Figures 5, 6). Conversely, HFD‐induced mitochondrial GCN5L1 depletion inhibits GRP75 acetylation on K567/612 to promote IP3R1‐GRP75‐VDAC assembly. The GCN5L1/GRP75 axis has been identified as a novel mechanism underlying HFD‐induced hepatic insulin resistance. Mitochondrial protein acetylation is highly associated with glucose and fatty acid metabolism, since the metabolic produced acetyl‐CoA is the substrate for protein acetylation. Protein acetylation in turn modulates enzyme activity and mitochondrial metabolism. Energy sensor AMPK‐mediated activation of MCU stimulates a rapid mitochondrial calcium transient to provide energy for mitosis.^[^ ^47^ ^]^ Thus, mitochondrial acetylation and calcium signaling are involved in energy sensing and metabolic adaptation. GRP75 possesses over 20 acetylated residues suggesting unknown functions of acetylation should be investigated.

In the current study, from the technical perspective, we were unable to detect HFD induced GRP75 acetylation levels and lysine residues in MAM fractions from livers or hepatocytes. Thus, whether HFD induced GRP75 acetylation promotes its translocation needs to be addressed in future work. Antibodies against specific GRP75 acetylation residues will be helpful to compare GRP75 acetylation in mitochondrial and MAM fractions to address the function of specific lysine acetylation in regulating its localization. In addition, the knockin mice with the GRP75 K567/612 mutation will be helpful to uncover the physiological role and regulatory pathways of GRP75 acetylation. Our data support that GRP75 K567/612 deacetylation enhances the interaction of GRP75 with IP3R1, however, GRP75‐2KR mutation shows moderate inhibition on insulin sensitivity in NCD mice or hepatocytes without palmitate treatment. Interestingly, HFD or PA treatment augmented this phenomenon. We observed higher GRP75 acetylation in HFD livers compared to NCD (data not shown). These findings suggest that global acetylation of GRP75 might contribute to its translocation. Additionally, mitochondrial GCN5L1 translocating to the cytosol is a critical step in HFD‐induced IR development, which specifically promotes K567/612 deacetylation of GRP75 to induce IP3R1‐GRP75‐VDAC complex assembly. Future studies are warranted to explore the critical factors driving mitochondrial versus cytosolic localization of GCN5L1. Elucidation of this mechanism may uncover a regulatory node underpinning mitochondrial retrograde signaling transduction. Hepatic deletion of GCN5L1 has been reported to increase fatty acid oxidation^[^ ^27^ ^]^ and lysosomal lipolysis.^[^ ^28^ ^]^ FAO provides plenty of acetyl‐CoA in mitochondria to facilitate protein acetylation, which could provide a non‐specific manner to promote global acetylation of GRP75. On the other hand, increased FAO or lipolysis in GCN5L1 LKO livers prevents lipid accumulation, which could contribute to insulin sensitizing.

The major feature of the IP3R‐GRP75‐VDAC complex in MAM is calcium transport, whose disorder induces ER stress and mitochondrial oxidative stress, which is considered as a major contributor to various diseases.^[^ ^47^ ^]^ However, we found acute ROS generation without mitochondrial damage does not induce IR (Figure S1, Supporting Information), which suggests mitochondrial/ER stress but not ROS level is more substantial for the development of IR. GRP75 belongs to the HSP family and binds to misfolded polypeptides assisting their refolding with HSP60, thus loss of GRP75 may activate UPR^MT^.^[^ ^48^ ^]^ Interestingly, UPR^MT^ induced by mitochondrial protease CLPP or LONP1 deletion protects mice from diet‐induced insulin resistance.^[^ ^17^, ^18^ ^]^ It's not clear whether acetylation regulates the chaperone function of GRP75 in the context of UPR^MT^ and whether this process participates in the regulation of insulin sensitivity.

Mitochondrial Ca^2^⁺ levels serve as key regulators of mitochondrial oxidative phosphorylation. Studies have shown that Ca^2^⁺ enhances the affinity of matrix dehydrogenases to their substrates, thereby potentially boosting ATP production.^[^ ^49^ ^]^ Notably, we observed increased ER‐mitochondrial calcium flux in GCN5L1‐deleted cells; however, these cells exhibited no significant alterations in mitochondrial respiration (Figure 3; Figure S1, Supporting Information). Emerging evidence suggests that Ca^2^⁺ may dictate the metabolic choice between glucose and fatty acid substrates.^[^ ^50^, ^51^ ^]^ Thus, it is plausible that the elevated mitochondrial calcium levels in GCN5L1‐deficient cells induce metabolic reprogramming rather than promoting OXPHOS activation, a hypothesis that could be validated by enhanced fatty acid oxidation (FAO) in the GCN5L1 LKO mouse model and GCN5L1‐KO HepG2 cells.^[^ ^27^, ^31^ ^]^ Meanwhile, high calcium levels decrease mitochondrial respiration, mediated by mPTP.^[^ ^52^ ^]^ Considering these aspects, the elevated mitochondrial calcium in GCN5L1 LKO hepatocytes did not lead enhanced oxidative phosphorylation.

Mitochondrial signaling transduction relies on mitochondrial metabolites release or mitochondrial protein translocation in response to stress, such as DELE1 and ATF5, whose mitochondrial targeting sequences (MTS) engage with mitochondrial membrane potential‐dependent proteases to initiate the translocation process.^[^ ^53^, ^54^ ^]^ GCN5L1 does not possess a canonical MTS but accumulates in hepatic mitochondria under NCD, while HFD or PA induced GCN5L1 translocating out of mitochondria may be due to increased surveillance of mitochondrial protein import under HFD or the mitochondrial calcium overload associated mitochondrial permeability transition pore (mPTP) open, which is a common way for mtDNA release. In addition, GRP75 possesses MTS, but is found to be localized in the cytoplasm, mitochondria, and MAMs.^[^ ^55^ ^]^ We identified that GRP75 is highly acetylated in livers, however, the function of GRP75 modification is mysterious and needs to be further investigated. Our data support the notion that mitochondrial GCN5L1 is critical in regulating specific protein acetylation, which makes mitochondrial homeostasis necessary for preventing IR development. These data implicate a novel mechanism about GRP75 acetylation involving in HFD‐induced hepatic insulin resistance. The pattern that mitochondria transfer signal outside in response to nutrient change is an interesting topic and worths to be further investigated. Although we demonstrate the exclusion of mitochondrial GCN5L1 in HFD‐fed mouse model, further investigation on GCN5L1 translocation and the underlying mechanism in human patients with type 2 diabetes and MASLD is needed to unveil the function of mitochondrial GCN5L1 in insulin sensitivity. Meanwhile, the antibody against GRP75‐K567/612 acetylation is needed to confirm the role of GRP75 acetylation in human patients with type 2 diabetes. The investigation will provide potential targets for drug design to treat type 2 diabetes.

## 4 Experimental Section

### Animal Studies

All animal protocols were in accordance with Institutional Guidelines and approved by the Animal Care and Use Committee of Tianjin Medical University. All mice used were C57BL/6J background and were kept under specific pathogen free (SPF) and temperature‐controlled environment with a 12‐hour light/dark cycle and housed with free access to water and normal chow diet.

GCN5L1 liver knockout mice (GCN5L1‐LKO) were described previously^[^ ^25^ ^]^ and compared to *flox/flox* littermate controls (provided by Dr. Michael Sack, NIH). GCN5L1 liver knockout mice were generated by crossing GCN5L1 flox/flox mice with albumin‐Cre transgenic mice. All mice were generated in the C57BL/6 background (cross with C57BL/6 mice over 10 generations). The mice were maintained on a 12‐hour light/dark cycle and housed three to five mice per cage with free access to water and a normal chow diet, unless indicated.

For the HFD model, all mice were started on a 60% high‐fat diet (HFD, D12492, Research Diets) at 8 weeks of age. For mice requiring AAV injections, the virus was injected at 6 weeks of age, followed by HFD feeding starting at 8 weeks, consistent with other groups.

Eight‐week‐old male Con was used, and GCN5L1‐LKO mice fed a high‐fat diet for 16 weeks to perform the following experiments: liver TEM (Figure 3b), blood glucose and plasma insulin measurements (Figure 4g), and i.p. insulin injection followed by phospho‐Akt (S473) protein expression analysis.

Six‐week‐old male wild‐type C57 mice were used and divided into three groups. Two groups received tail vein injections of AAV‐TBG‐eGFP and were fed either a normal chow or a high‐fat diet starting two weeks post‐injection. The third group received AAV‐TBG‐MtG injections and began HFD feeding two weeks later. ITT were performed at 8 weeks of HFD feeding (Figure 4i). After 16 weeks of HFD, mice were sacrificed for liver collection. Subcellular fractions of hepatocytes were isolated via ultracentrifugation, and GRP75/GCN5L1 protein levels were analyzed by Western blotting (Figure 6a–c).

In addition, 6‐week‐old male C57 wild‐type mice were injected with either AAV‐GRP75‐2KR or AAV‐GRP75‐2KQ in the tail vein. Two weeks after injection, the mice were placed on a high‐fat diet for 2 weeks. Subsequently, ITT and expression of ER stress‐related genes were assessed. Subsequently, ITT and expression of ER stress‐related genes were assessed (Figure 6i,j).

For hepatic overexpression experiments, 2 × 10^11^ genome copies/mouse of AAV in 100 µL saline were administered via tail vein injection to 6–8 weeks old male mice. Insulin tolerance tests (ITTs) were performed with GRP75 mutant‐injected mice 3 weeks after AAV injection or after 2 weeks of HFD feeding. Bodyweight (0.75 U kg^−1^) of insulin solution was used. Plasma insulin levels were measured by a mouse insulin immunoassay kit (EZassay).

### Cell Culture Studies

Primary hepatocytes were isolated from GCN5L1*^flox/flox^*, GCN5L1*^flox/flox^*‐Albumin*^Cre^* mice at the age of 8—12 weeks following the previous protocol.^[^ ^25^ ^]^ Hepatocytes were cultured in DMEM medium containing 10% FBS and 1% p/s.

HepG2 (Cat# HB‐8065, RRID: CVCL_0027) and HEK293T (Cat# CRL‐3216, RRID: CVCL_0063) cells were obtained from the American Type Culture Collection (ATCC). All cells were maintained in high‐glucose Dulbecco's Modified Eagle's Medium (DMEM) supplemented with 10% fetal bovine serum (FBS) and incubated at 37 °C in a humidified chamber containing 5% CO_2_.HepG2 cells were PA‐treated to induce insulin resistance, followed by assessment of GCN5L1 expression. Stable HepG2 cell lines with targeted gene knockouts or overexpression were established via lentiviral infection. HEK293T cells were primarily utilized for Co‐IP assays and the packaging of lentivirus and adeno‐associated virus.

### Mitochondrial Peroxide Assay

Primary hepatocytes were seeded into 96‐well black/clear bottom plates at a density of 10 000 cells per well. After attachment, cells were stained with MitoSOX (Beyotime) and Hoechst 33 342 (MCE) or treated with compounds before staining. After staining, the medium was replaced with fresh PBS, and fluorescence was measured using a microplate reader. Results were standardized to the fluorescence intensity of Hoechst 33 342.

### Antibodies and Reagents

Antibody against GCN5L1 was a Gift from Dr. Michael Sack (NIH/NHLBI). The pY, IR, p‐Akt^ser473^, Akt, CHOP, GRP75, ACK, VDAC, IP3R1, and MFN2 antibodies were purchased from Cell Signaling Technology. Antibodies against β‐actin, Tubulin, Myc, GLUD1, His, Cytochrome C, and TOM20 were from ABClonal. Antibody against the Flag tag was purchased from Sigma. The FACL4 antibody was from Abcam. The MCU antibody was from Proteintech. Antibody against HSP60 was purchased from Santa Cruz (USA). The detailed information on antibodies is listed in Table S3 (Supporting Information).

Palmitic acid (PA), Acetyl‐CoA, Antimycin A, Rotenone, Oligomycin, FCCP, Saponin, and Benzonase were purchased from Sigma. rProtein A/G MagBeads (IP Grade), Liposomal Transfection Reagent, and qPCR SYBR Green were from Yeasen. Glycogen Periodic Acid Schiff (PAS) Stain Kit, proteinase K, and puromycin were purchased from Solarbio. Thapsigargin (Tg) was from Abcam. ATP and BiP inducer X were from MCE. 4‐PBA was from Selleck. The detailed information on the reagents is listed in Table S4 (Supporting Information).

### Plasmids Construction and Transfection

The coding sequence of GRP75, VDAC1, GCN5L1, IP3R1 and relative fragments were amplified from mouse cDNA and cloned into the pLenti‐CMV vector containing 3×FLAG or MYC tag at C‐terminal. GRP75 mutants were generated using a site‐directed mutagenesis kit. The constructed plasmids were transferred into HEK‐293T or HepG2 cells using a liposomal transfection reagent (Yeasen). The lentiviral sgGCN5L1 (sequence: GAGGTCCATACCCCACATTG) was generated as previously described.^[^ ^56^ ^]^ The detailed information of plasmid construction is listed in Table S5 (Supporting Information).

### RNA Interference

All siRNA oligonucleotides were designed (Table S6, Supporting Information) and purchased from GenePharma Company. Cells were transfected with siRNA using Lipofectamine RNAiMAX following the manufacturer's instructions.

### Subcellular Fractionation

Crude mitochondrial fraction was isolated as previously described.^[^ ^25^ ^]^ Purified MAM and mitochondrial fractions were performed following a Nature Protocol.^[^ ^33^ ^]^ MAM and mitochondrial proteins were separated using saponin for western blot analysis. Briefly, crude mitochondrial fractions were incubated with 1 mg mL^−1^ of saponin in isolation buffer (225 mm mannitol, 75 mm sucrose, 0.5% BSA, 0.5 mm EGTA and 30 mm Tris‐HCl pH 7.4) for 15 min, followed by centrifuge at 12 000 rpm for 10 min at 4 °C. MAM proteins were in supernatant, and pellets were lysed in RIPA buffer as mitochondrial fraction. Related antibodies were used as markers (FACL4: MAM; GDH: mitochondrial matrix; cytochrome C: intermembrane space) to indicate relative fractions. An alternative method for identifying protein localization was performed by incubation of crude mitochondrial protein with proteinase K (10 µg of proteinase K mg^−1^ of mitochondrial protein) with/without triton X‐100 (2 mg of Triton X‐100/mg of mitochondrial protein) at RT for 1 h,^[^ ^57^ ^]^ followed by subjecting to immunoblotting.

### Adeno Virus and Adeno‐Associated Virus Production

Mitochondrial‐restricted GCN5L1 (MtG), GRP75 mutants, and eGFP were subcloned into the AAV‐TBG vector. AAVs were produced according to the protocol.^[^ ^58^ ^]^ First, HEK293T cells were transfected with three plasmids using PEI: i) pAAV, which contained the rAAV genome of interest; ii) pUCmini‐iCAP‐PHP, which encoded the viral replication and capsid proteins; and iii) pHelper, which encoded adenoviral proteins necessary for replication. Using this triple‐transfection approach, a single‐stranded rAAV genome was packaged into an AAV‐PHP capsid in HEK293T cells. AAV‐PHP viruses were then harvested, purified, and tittered by quantitative PCR (qPCR). Purified viruses were intravenously delivered to mice via tail vein injection, and gene expression was later assessed using molecular, histological, or functional methods relevant to the experimental aims. The adeno viruses expressing MtG, GCN5L1, or eGFP were generated by GeneChem.

### Immunoprecipitation and IP Coupled Mass Spectrometry

Plasmids coding relative genes were transfected into HEK‐293T cells using a liposomal transfection reagent (Yeasen). Thirty‐six hours after transfected, cells were lysed with Co‐IP lysis buffer (20 mm Tris‐HCl, pH 8.0, 137 mm NaCl, 1% Nonidet P‐40, 2 mm EDTA, PMSF, NAM, proteinase inhibitor, and TSA cocktails) and proteins were extracted from the supernatant of the lysate after 10 min centrifuging at 4 °C, 12 000 rpm. Antibodies were incubated with supernatant for 24 h. Protein A/G beads (Yeasen) were added and incubated with supernatant for an extra 4 h. The beads were then washed with Co‐IP wash buffer (10 mm Tris base, pH 7.4, 1 mm EDTA, 1 mm EGTA, pH 8.0, 150 mm NaCl, 1% Triton X‐100) for 3 times using a magnetic particle separator. The supernatant fraction was boiled with 4× SDS loading buffer for western blotting. AAV‐mitochondria‐GCN5L1 or AAV‐GFP were injected into WT mice through the tail vein. Mitochondria‐GCN5L1 was accumulated from liver mitochondrial lysate by flag resin. The immunoprecipitation samples were used for mass spectrometry analysis as previously described.^[^ ^59^ ^]^

### Acetyl‐Proteomic Analysis of Mitochondrial Protein

The analysis was done as previously described.^[^ ^26^ ^]^ eGFP or mitochondrial‐restricted GCN5L1 expressing adeno virus was injected into wildtype mice through the tail vein (5 × 10^8 ^pfu mouse^−1^). Isolated liver mitochondria were analyzed from these mice. Triplicate samples (400 µg each) were sequentially reduced, alkylated, digested overnight with trypsin, and labelled with 6‐plex Tandem Mass Tag (TMT) reagents (Thermo Fisher Scientific, San Jose, CA). Six labelled protein digests were pooled and desalted. 5% of the total digest was separated into 12 fractions using high‐pH reverse phase liquid chromatography (bRPLC). Each fraction was analyzed on an Orbitrap Fusion Lumos (Thermo Fisher Scientific) based nano‐LCMS system for protein‐level quantification. The residual 95% was incubated with anti‐acetyl‐lysine antibody agarose beads (ImmuneChem, Burnaby British Columbia Canada) to enrich for acetyl‐lysine peptides as described. Eluted peptides from the IP enrichment were separated into 8 fractions using bRPLC and analyzed on by nano‐LCMS for acetyl peptide identification and quantification. Specifically, bRPLC fractions were separated on an EasySpray column (Thermo Fisher ES803; 50 cm by 75 µm packed with PepMap RSLC C18 2 µm particles) using a 90 min linear gradient of 5% to 35% CAN‐0.1% formic acid at a flow rate of 300 nL min^−1^. Mass analysis was carried out in data‐dependent mode, where MS1 scans at 60 000 mass resolution were carried out with the full MS range from m/z 375 to 1500 and 10 higher‐energy collisional dissociation (HCD) MS2 scans at 38 HCD energy and 30 000 resolution.

Peptide and protein identification and quantification were carried out on the Proteome Discoverer 2.2 platform (PD 2.2, Thermo Fisher Scientific). For protein‐level, search settings were 10 ppm at MS1 and 20 ppm at MS2, cysteine carbamidomethylation and TMT label on N‐termini and lysine as fixed modification, methionine oxidation as variable modification, and 1 tryptic miscleavage. For acetylated peptides, search settings were 10 ppm at MS1 and 20 ppm at MS2, cysteine carbamidomethylation and TMT label on N‐termini as fixed modification, methionine oxidation and lysine TMT‐label and acetylation as variable modifications, and 4 tryptic mis‐cleavages.

Over 7600 acetyl‐lysine peptides were detected with < = 1% false discovery rate in the IP‐enriched dataset. The acetyl‐lysine peptides’ quantitative values were normalized using normalization factors that were calculated based on the total report ion intensities of their corresponding channels from the total protein data set.

### Calcium Imaging

Primary hepatocytes were isolated from Con, GCN5L1 LKO, and LKO with AAV‐GRP75‐2KR and AAV‐GRP75‐2KQ expression mice. Cells were then seeded onto collagen‐I‐coated 35 mm confocal dishes and incubated at 37 °C for 4 h. Cells were transfected with the [Ca^2+^]_mt_ sensor plasmid pCAG mito‐RCaMP1h (Addgene #105 013), pCAG mito‐RCaMP1h‐IRES‐MtG or [Ca^2+^]_cyto_ sensor plasmid pGP‐CMV‐jGCaMP7f (Addgene #104 483) or pGP‐CMV‐jGCaMP7f‐IRES‐MtG. After 48 h, hepatocyte [Ca^2+^]_mt_ or [Ca^2+^]_cyto_ was monitored by LSM 900 confocal microscope (Zeiss). Before the experiment, cell culture media were removed and the cells were washed twice with calcium‐free D‐HBSS (Pricella), then fresh calcium‐free buffer was used for calcium imaging assays. Cells were treated with Tg or ATP at the indicated concentrations to induce Ca^2+^ signaling. Images were captured with a 20× objective at the following excitation/emission wavelengths: 561 nm/593 ± 46 nm for mito‐RCaMP1h and 488 nm/510 nm for jGCaMP7f. Images were recorded right after the injection of Thapsigargin (Tg) or ATP at 5‐second intervals, for a total of 200 pictures. The quantification was determined by normalizing the fluorescence of single cell to its starting point.

The area under the curve (AUC) for total calcium excursion was calculated using the trapezoidal rule. Calcium levels were integrated over time, with baseline subtraction (*t* = 0 min) to quantify total calcium excursion.

### Immunoblotting

An equal amount of protein samples ≈50–80 µg was loaded into the SDS‐PAGE gel respectively and subjected to electrophoresis. Separated proteins were then transferred onto a methanol‐activated polyvinylidene fluoride (PVDF) membrane (MF‐Millipore). The membrane was blocked with 10% skim milk in PBS containing 0.05% Tween‐20 (PBS‐T). Primary antibodies were added and incubated with the blocked membrane at 4 °C overnight. The membrane was then incubated with anti‐mouse Ig‐G HRP or anti‐rabbit Ig‐G HRP secondary antibodies.

### Protein Purification

His tagged proteins were expressed in BL21 bacterial cells induced with isopropyl β‐D‐1‐thiogalactopyranoside, purified using Ni‐NTA Beads 6FF, and then washed with wash buffer (20 mm Na_3_PO_4_, pH 7.4, 500 mm NaCl, and 20 mm Imidazole), and eluted with wash buffer containing 500 mm Imidazole. The protein samples were concentrated by ultrafiltration and stored in protein storage buffer (20 mm Tris‐HCl, pH 8.0, 100 mm NaCl, 10% Glycerol) at −80 °C.

### In Vitro Acetylation Assay

GRP75 and GCN5L1 proteins were purified from BL21, 25 µg GRP75 was incubated in 50 µL reaction buffer (25 mm Tris‐HCl, pH 8.0, 50 mm KCl, and 1 mm dithiothreitol) with the indicated dose of acetyl‐CoA at 37 °C for 3 h, then the samples were subjected for western blot analysis.

### Seahorse Analysis

Seahorse assays were performed according to the manufacturer's protocol. In brief, primary hepatocytes were seeded in an XF24 plate at 3 × 10^4 ^cells well^−1^, the experimental process followed the manufacturer's instructions with the injection of port A (1.25 mg mL^−1^ Oligomycin), port B (1 µm FCCP), port C (1 µm antimycin A and 1 µm Rotenone).

### MAM Quantification

Quantification of MAM was performed based on published protocols^[^ ^7^ ^]^ with minor modifications. The TEM images were analyzed using ImageJ. The mitochondrial and ER membranes were delineated using the freehand tool. The selected areas were converted to masks, and the total number, area, length of ER were calculated. For the acquisition of MAM quantification, the length of ER connected to mitochondria was normalized to total ER length.

### mRNA Expression Profiling from Public Data

Hepatic *GCN5L1* gene expression analysis was performed using the publicly available NCBI GEO dataset (www.ncbi.nlm.nih.gov/geo, GSE15653 and GSE 23 343). Within the dataset, a lean control group (control, *n* = 5 or *n* = 7) and a group of patients with T2D (T2D, *n *= 9 or *n *= 10) were selected, and GCN5L1 mRNA expression levels were evaluated between the groups.

### Blue‐Native PAGE Analysis

Primary hepatocytes isolated from mice were lysed in an ice‐cold buffer containing digitonin (2%, v/v), Bis‐Tris (50 mm), NaCl (50 mm), glycerol (10%, v/v) and protease inhibitors, at pH 7.4, for 30 min on ice. The lysates were then centrifuged at 17 000 g for 20 min at 4 °C to remove insoluble debris. The resulting supernatant containing the protein complexes was mixed with BeyoGel Blue Native PAGE Sample Buffer (Beyotime) and loaded onto BeyoGel Blue Native PAGE 4–13% gradient precast gels (Beyotime). Electrophoresis was performed at 4 °C. Subsequent analysis was performed via either Coomassie blue staining or antibody incubation.

### Statistical Analysis

GraphPad Prism 9 was used for data analysis. A comparison between two selected groups was performed using a two‐tailed unpaired Students *t*‐test or one‐way analysis of variance. A *p*‐value < 0.05 was considered statistically significant (*p < 0.05, **p < 0.01, and ***p < 0.001). For GSE data analysis, Kolmogorov–Smirnov and Shapiro–Wilk tests were used to assess data normality. The data were confirmed to follow a normal distribution, thereby justifying the use of *t*‐tests. The data represented the mean ± standard error of the mean. Three or more independent experiments were performed in all experiments. Sample sizes were chosen to satisfy statistical power based on previous experience.

## Conflict of Interest

The authors declare no conflict of interest.

## Author Contributions

D.W. and J.Z. contributed equally to this work. This study was conceived by L.W. and L.Z. D.W., J.Z., Y.W., L.W., and L.Z. contributed to the study conception and design. Y.C., Y.W., and M.S. were consultants, provided important suggestions and manuscript editing. Experiments were performed by D.W., J.Z., X.Y., Q.Z., X.H., X.L., H.L., and X.B. Data were analyzed and interpreted by D.W. and J.Z. Proteomic data were analyzed by X.B. and K.Z. The manuscript was written by D.W., M.S., Y.W., L.W., and L.Z. with input from all authors.

## Supporting information



Supporting Information

Supplemental Table 1

Supplemental Table 2

Supplemental Table 3

Supplemental Table 4

Supplemental Table 5

Supplemental Table 6

## Data Availability

The data that support the findings of this study are available from the corresponding author upon reasonable request.
