# Supplementary material for: Acetylation of the Mitochondrial Chaperone GRP75 Governs ER‐Mitochondrial Calcium Homeostasis and Hepatocyte Insulin Resistance
Source: Adv Sci (Weinh). 2025 Sep 26;12(46):e08991. doi: 10.1002/advs.202508991 (PMC12697862; doi:10.1002/advs.202508991)
Supplement: Supplementary file 1 — Supporting Information [file ADVS-12-e08991-s001.docx]

**Supplementary Fig. 1. Mitochondrial GCN5L1 modulates hepatocyte insulin signaling and ER stress.
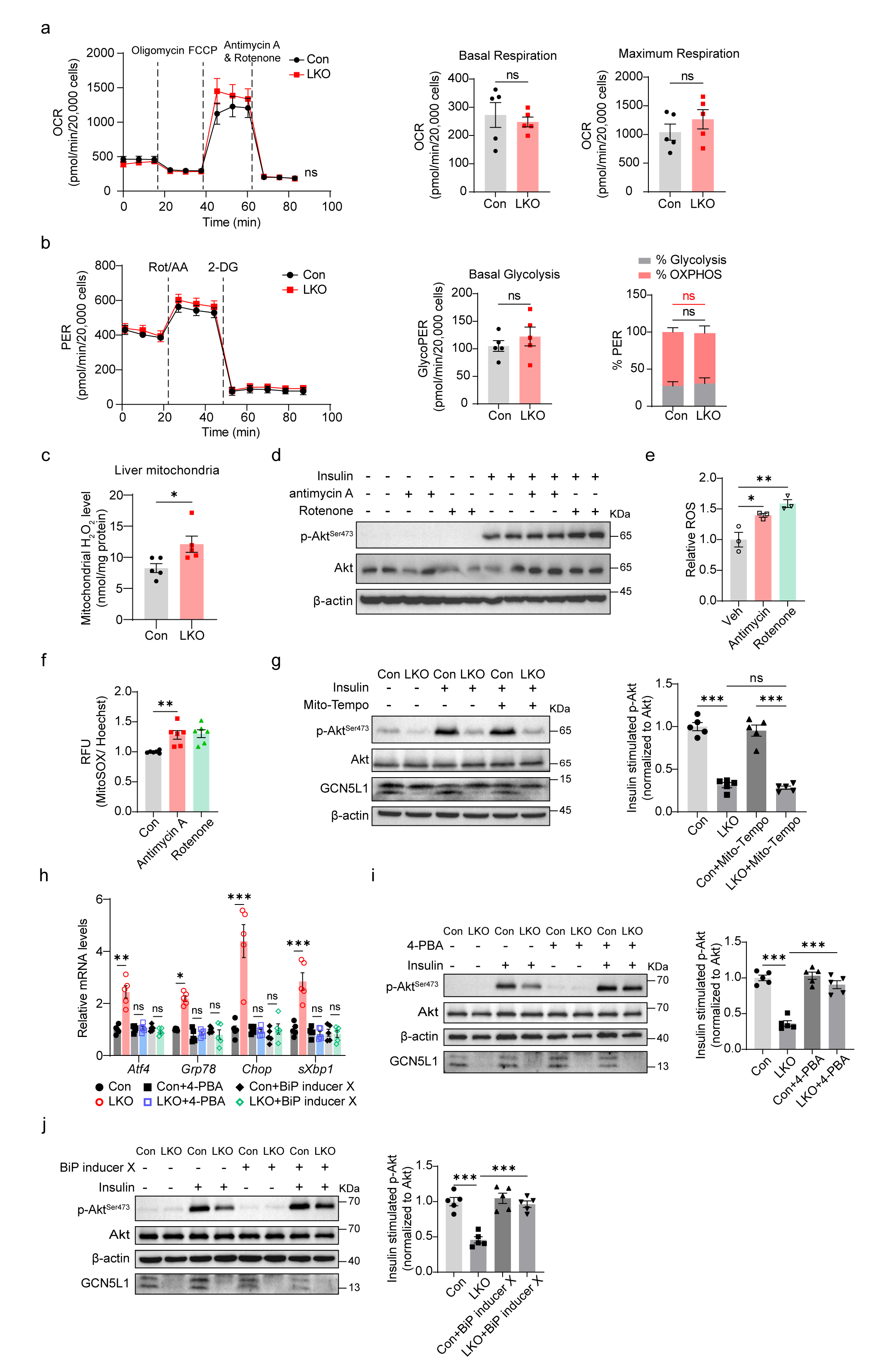
**

1. OCR was measured by Seahorse using primary hepatocytes from GCN5L1 LKO and controls (left panel). Quantification of basal respiration and maximum respiration (right panels). *n=5*.
2. Seahorse XF Glycolytic rate assay determines proton efflux rate (PER) under basal conditions, after inhibiting oxidative phosphorylation with rotenone/ antimycin A (Rot/AA), and after inhibiting glycolysis with 2-DG. A PER trace from the experiments (Left), basal glycolytic PER (Mid), percentage of PER from glycolysis versus oxidative phosphorylation (Right). *n=5*

(c) Mitochondrial H_2_O_2_ levels were measured using isolated mitochondria from liver tissues of GCN5L1 LKO and control mice. *n=5*.

(d-f) Hepatocytes were pretreated with ETC inhibitors (antimycin A, 50 nM; Rotenone, 10 nM) for 10 min, followed by incubation with insulin (10 nM, 15 min). Cell lysates were subjected to western blotting (d), cellular ROS levels were analyzed with DCFDA staining and flowcytometry analysis (e) and mitochondrial peroxidation levels were analyzed with MitoSOX and Hochest staining and Microplate reader analysis (f).

(g) Hepatocytes from GCN5L1 LKO and controls were pretreated with Mito-Tempo (5 μM) for 12 h, followed by incubation with insulin (10 nM, 15 min). Cell lysates were subjected to western blotting, p-Akt levels were normalized to Con with insulin treatment. *n=5*.

(h) Primary hepatocytes from GCN5L1 LKO and controls were treated with 4-PBA (5 mM, 24h) or Bip inducer X (5 μM, 24h), and the expression of ER stress-related genes was analyzed by qPCR. *n=5*.

(i-j) Primary hepatocytes from GCN5L1 LKO and controls were pretreated with 4-PBA (5 mM, 24h) or Bip inducer X (5 μM, 24h), followed by incubation with insulin (10 nM, 15 min). Cell lysates were subjected to western blotting, p-Akt levels were normalized to Con with insulin treatment. *n=5*.

All values are expressed as means ± SEM. **P  <  0.05, **P  <  0.01, ***P  <  0.001*. ns, not significant. Statistical analyses were performed using two-tailed unpaired Student’s t-test (a, b and c), one-way ANOVA with multiple comparisons (e-g, i and j)，or two-way ANOVA with multiple comparisons (h)


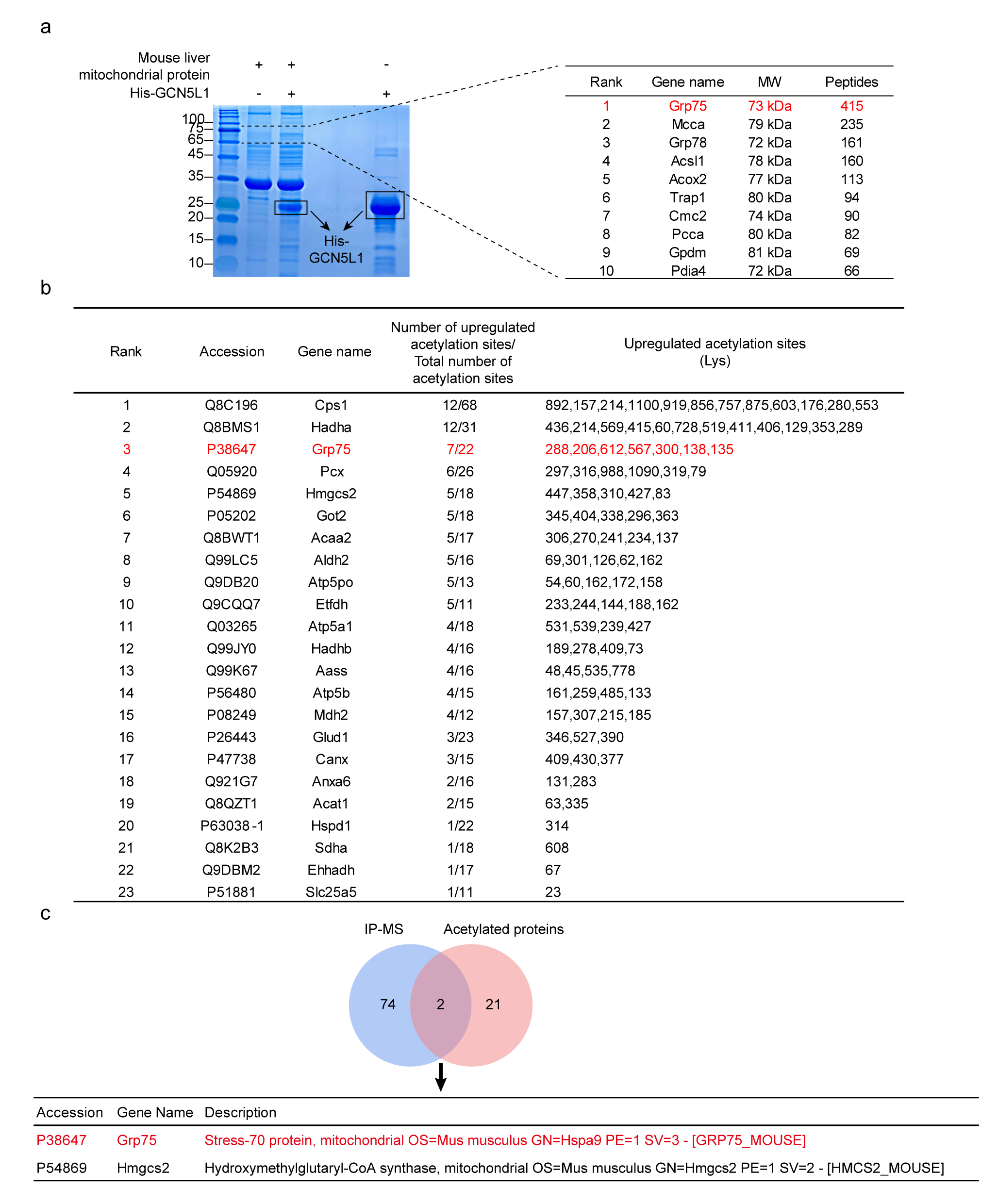


**Supplementary Fig. 2. Proteomic screenings to identify target(s) which interact with GCN5L1 and show changes in protein acetylation.**

1. GCN5L1 protein was purified from BL21 and incubated with liver mitochondrial extract. Coomassie blue stained gel indicated a differentiated band that was subjected to MS analysis. The top 10 identified proteins are shown on the right.
2. Top 23 acetylated proteins from acetyl-proteomic data, indicating total number of acetylated lysine residues and the ones significantly regulated by mitochondrial GCN5L1.
3. Intersection of IPMS and acetyl group data. Gene name and description are shown in the table.


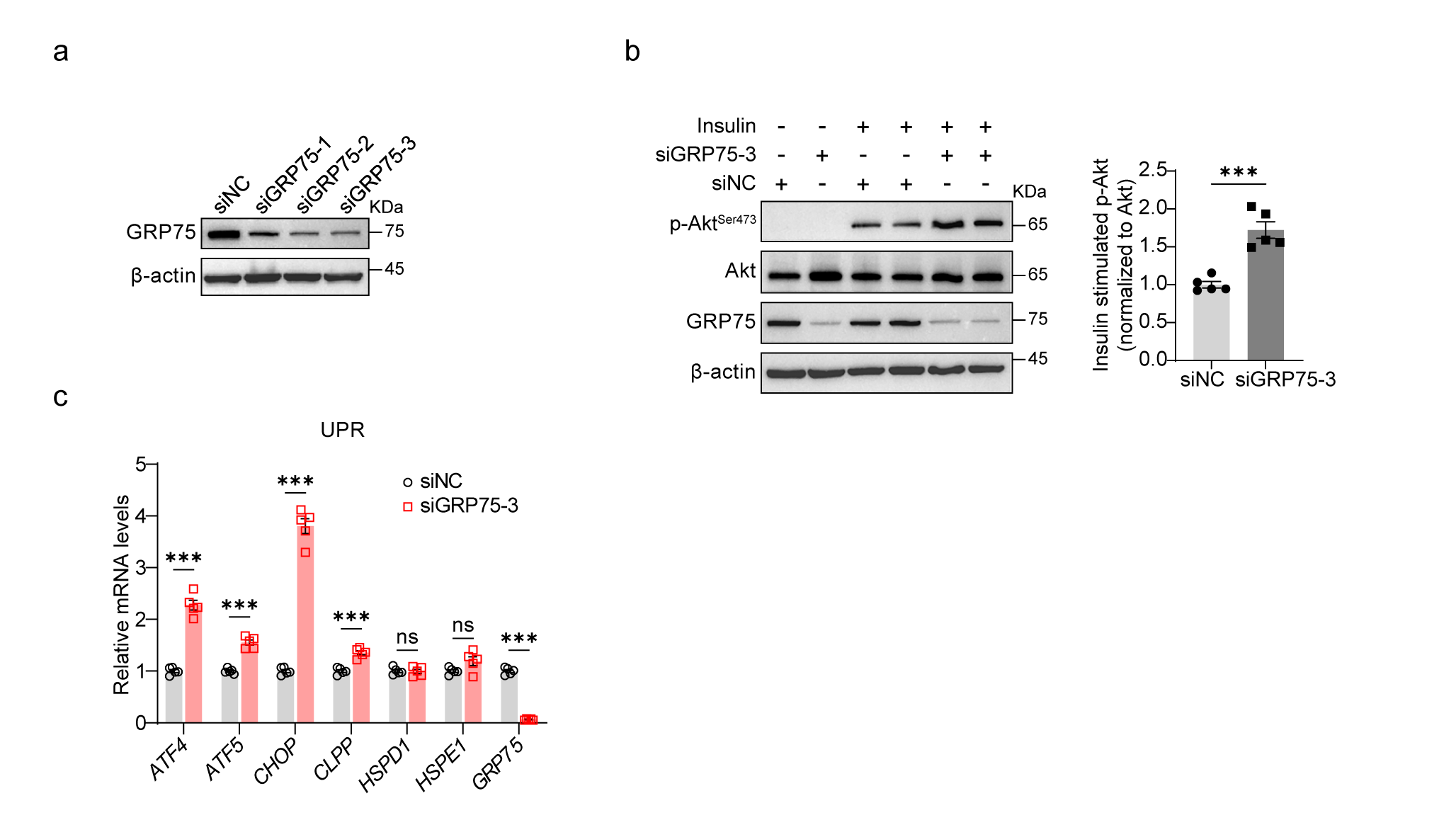


**Supplementary Fig. 3. Knockdown of GRP75 increases UPR^MT^ and insulin signaling.**

1. Immunoblotting image indicates GRP75 knockdown efficiency in HepG2 cells. siGRP75-3 was used for further experiments.
2. Immunoblotting image shows insulin signaling in GRP75 knockdown HepG2 cells. p-Akt levels were analyzed upon insulin stimulation. *n=5*.
3. RT-PCR analysis of mitochondrial stress and UPR^MT^ related gene expression in HepG2 cells. *n=5*.

All values are expressed as means ± SEM. **P  <  0.05, **P  <  0.01, ***P  <  0.001*. ns, not significant. Statistical analyses were performed using two-tailed unpaired Student’s t-test (b), or two-way ANOVA with multiple comparisons (c).


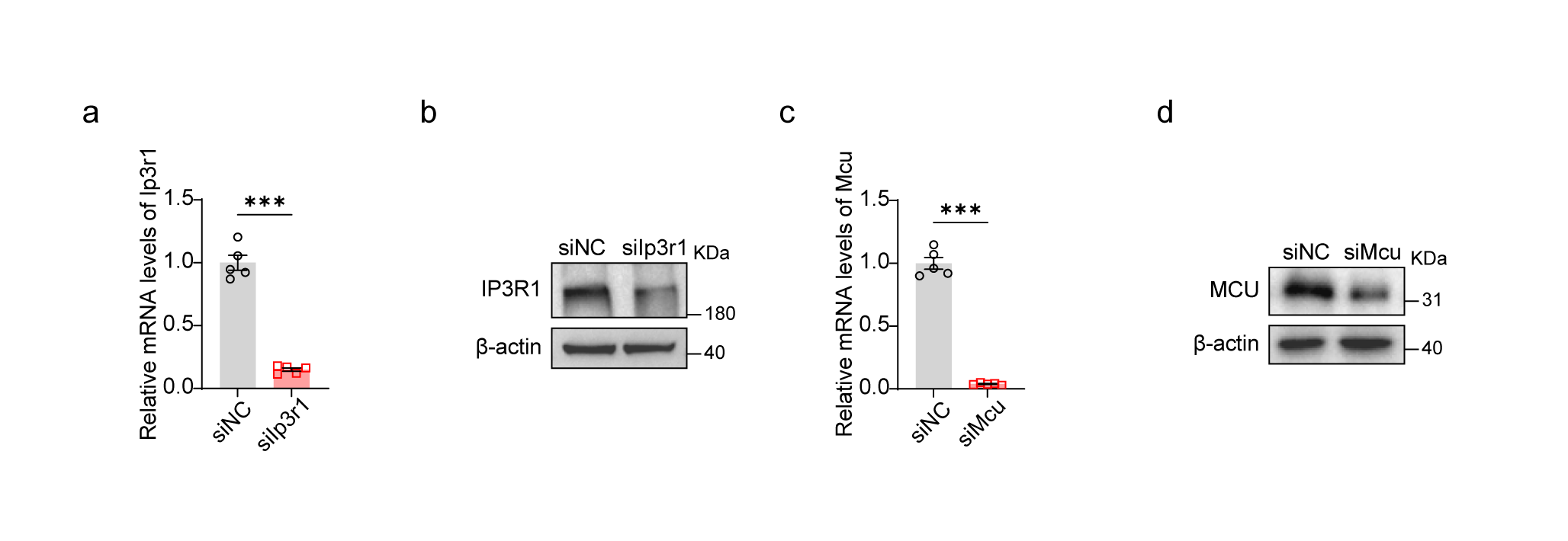


**Supplementary Fig. 4.**

(a-b) Knockdown efficiency of IP3R1 in hepatocytes with siRNA transfection. IP3R1 expression levels were evaluated by qPCR (a, *n=5*) and immunoblotting (b).

(c-d) Knockdown efficiency of MCU in hepatocytes with siRNA transfection. MCU expression levels were evaluated by qPCR (c, *n=5*) and immunoblotting (d).

All values are expressed as means ± SEM. **P  <  0.05, **P  <  0.01, ***P  <  0.001* by two-tailed unpaired Student’s t-test.


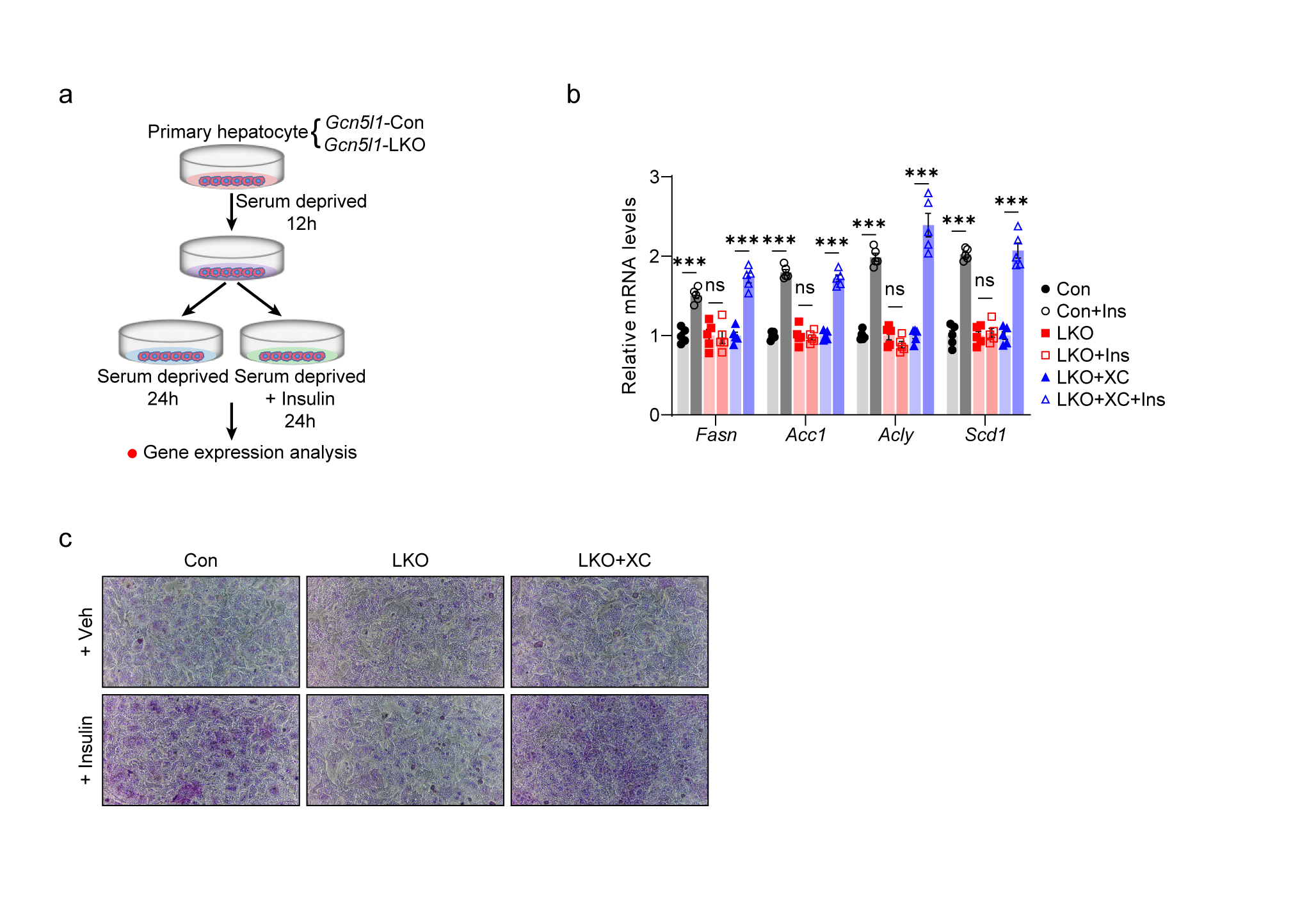


**Supplementary Fig. 5. GCN5L1 deletion reduces insulin action in primary hepatocytes through disturbance of ER-mitochondrial calcium homeostasis**

1. Illustration of experimental strategy in b.
2. Relative mRNA levels of lipogenic related genes in hepatocytes with treatment of insulin (10 nM). *n=5*.
3. PAS staining of glycogen in hepatocytes with indicated genotype and treatment.

All values are expressed as means ± SEM. **P  <  0.05, **P  <  0.01, ***P  <  0.001*. ns, not significant. Data were analyzed by two-way ANOVA with multiple comparisons.


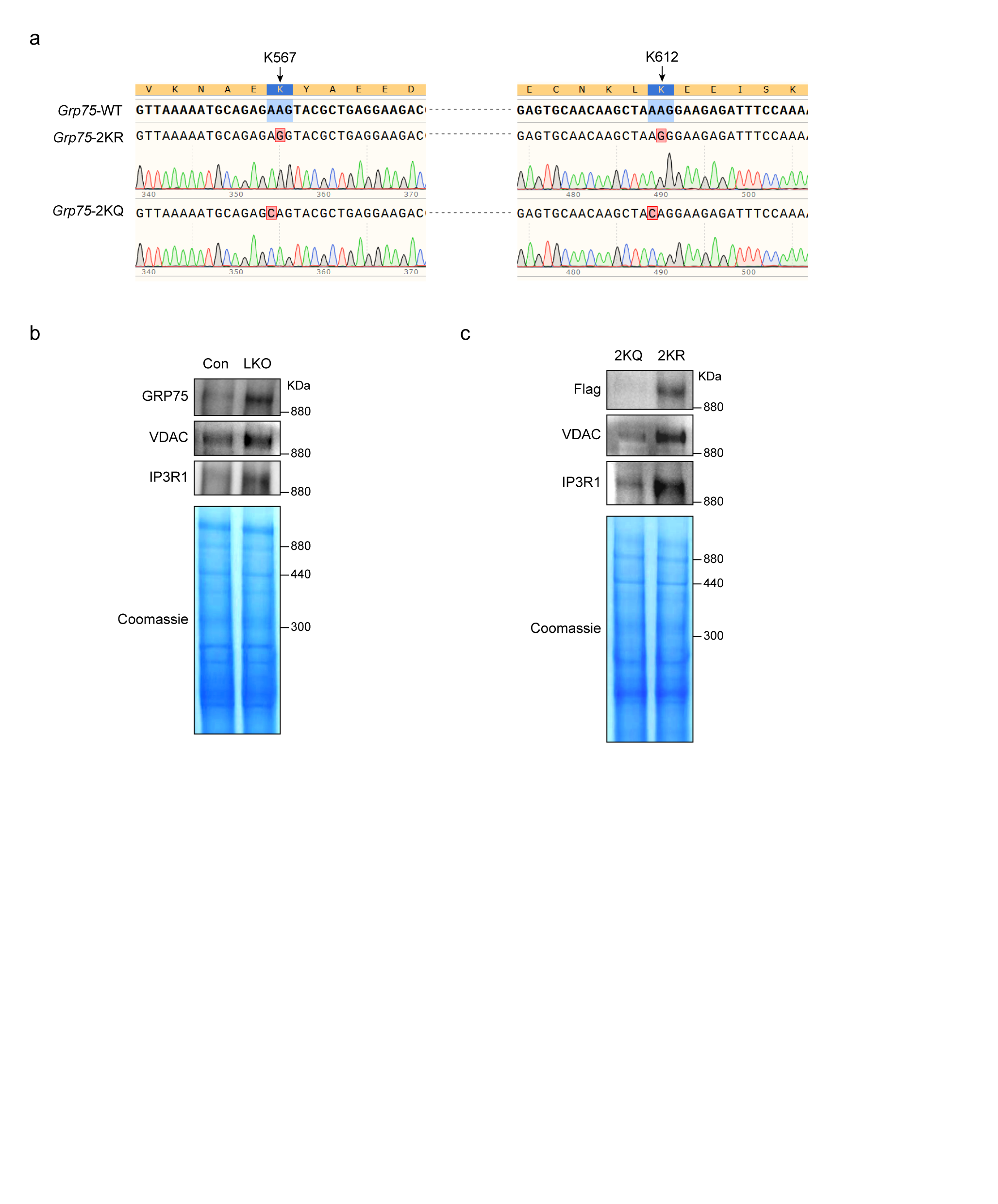


**Supplementary Fig. 6.**

1. DNA sequencing to confirm *Grp75*-2KR and 2KQ.
2. Blue-native PAGE was used to analyze IP3R1-GRP75-VDAC complex using primary hepatocytes from control and GCN5L1 LKO mice.
3. Blue-native PAGE was used to analyze IP3R1-GRP75-VDAC complex using primary hepatocytes from AAV-GRP75-2KQ/2KR mice.


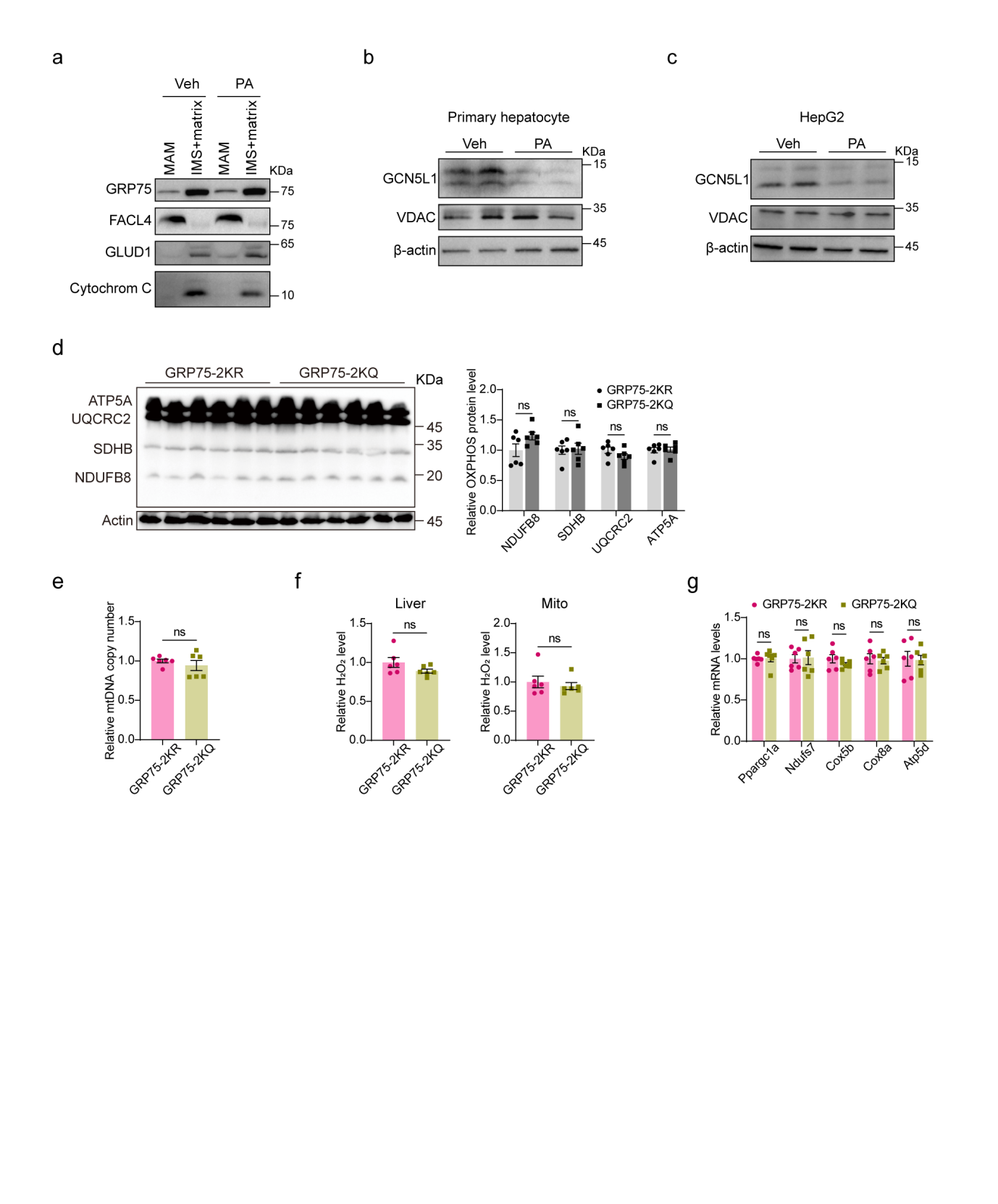


**Supplementary Fig. 7 Related to Figure 6**

1. MAM and mitochondrial proteins were isolated from hepatocytes with/without PA treatment. GRP75 localization was analyzed by immunoblotting.

(b-c) Mitochondrial proteins were isolated from hepatocytes and HepG2 cells with/without PA treatment and subjected to immunoblotting.

(d) Immunoblotting of OXPHOS complexes in livers of wildtype mice with AAV-GRP75-2KR and AAV-GRP75-2KQ expression. NDUFB8, SDHB, UQCRC2 and ATP5A levels were normalized to β-actin. *n=6*.

(e) Quantification of mtDNA copy number by qPCR in livers of wildtype mice with AAV-GRP75-2KR and AAV-GRP75-2KQ expression. *n=6*

(f) H_2_O_2_ levels in liver homogenates and isolated mitochondria were measured from AAV-GRP75-2KR and AAV-GRP75-2KQ-overexpressing mice. *n=6*.

(g) Relative mRNA levels of mitochondrial biogenesis related genes in livers of wildtype mice with AAV-GRP75-2KR and AAV-GRP75-2KQ expression. *n=6*.

All values are expressed as means ± SEM. ns, not significant. Statistical analyses were performed using two-way ANOVA with multiple comparisons (d and g), or two-tailed unpaired Student’s t-test (e and f).
